# *PAXX*, Not *NHEJ1* Is an Independent Prognosticator in Colon Cancer

**DOI:** 10.3389/fmolb.2020.584053

**Published:** 2020-10-23

**Authors:** Mohit Arora, Sarita Kumari, Jay Singh, Anita Chopra, Shyam S. Chauhan

**Affiliations:** ^1^Department of Biochemistry, All India Institute of Medical Sciences, New Delhi, India; ^2^Laboratory Oncology Unit, Dr. BRA-IRCH, All India Institute of Medical Sciences, New Delhi, India

**Keywords:** PAXX, NHEJ1 gene, colon cancer, The Cancer Genome Atlas, DNA methylation, DNA Repair

## Abstract

Classical Non-homologous End Joining (NHEJ) pathway is the mainstay of cellular response to DNA double strand breaks. While aberrant expression of genes involved in this pathway has been linked with genomic instability and drug resistance in several cancers, limited information is available about its clinical significance in colon cancer. We performed a comprehensive analysis of seven essential genes, including *XRCC5*, *XRCC6*, *PRKDC*, *LIG4*, *XRCC4*, *NHEJ1*, and *PAXX* of this pathway, in colon cancer using multi-omics datasets, and studied their associations with molecular and clinicopathological features, including age, gender, stage, *KRAS* mutation, *BRAF* mutation, microsatellite instability status and promoter DNA methylation in TCGA colon cancer dataset. This analysis revealed upregulation of *XRCC5*, *PRKDC*, and *PAXX* in colon cancer compared to normal colon tissues, while *LIG4* and *NHEJ1* (XLF) displayed downregulation. The expression of these genes was independent of age and *KRAS* status, while *XRCC5*, *PRKDC*, and *LIG4* exhibited reduced expression in *BRAF* mutant tumors. Interestingly, we observed a strong association between *XRCC6*, *XRCC5*, *PRKDC* and *LIG4* overexpression and microsatellite instability status of the tumors. In multivariate analysis, high *PAXX* expression emerged as an independent prognostic marker for poor overall and disease specific survival. We also observed hypomethylation of *PAXX* promoter in tumors, which exhibited a strong correlation with its overexpression. Furthermore, *PAXX* overexpression was also associated with several oncogenic pathways as well as a reduction in numbers of tumor-infiltrating lymphocytes.

## Introduction

Colorectal cancer (CRC) is the fourth most commonly diagnosed cancer and the third most common cause of cancer related deaths worldwide (Bray et al., 2018; Rawla et al., 2019). It represents a group of heterogeneous diseases that are characterized by a range of genomic and epigenomic alterations (The Cancer Genome Atlas Network, 2012). The knowledge regarding the molecular landscapes of CRCs is rapidly increasing, which has led to advancements in early detection methodologies and hence reduction of mortality rates (Arnold et al., 2017).

DNA double strand breaks (DSBs) are inherently induced during several physiological conditions, including stem cell differentiation, cell division, autophagy, and senescence. Homologous recombination repair (HRR), classical non-homologous end joining (c-NHEJ or NHEJ) and alternative end joining (Alt-EJ) are the three DNA damage repair systems, which efficiently repair DSBs, and thus maintain genomic stability during physiological stress (Chang et al., 2017). HRR functions by using a homologous DNA strand as a template to perform error free repair at DSB sites. Contrary to this, NHEJ is the primary DNA damage repair pathway and perform template independent repair of deleterious DSBs (Chang et al., 2017). Alt-EJ is a less characterized mechanism which works as a backup for both HRR and NHEJ in case of excessive DNA damage, and also utilizes micro-homologies between distant DNA sites for template dependent repair.

The core c-NHEJ system consists of Ku70/80 heterodimer (encoded by *XRCC6* and *XRCC5*, respectively), XRCC4, DNA-dependent protein kinase catalytic subunit (DNA-PKcs, encoded by *PRKDC*), DNA Ligase 4 (encoded by *LIG4*), and *XRCC4*-like factor (XLF, encoded by *NHEJ1*). DNA damage sites are quickly recognized by Ku70/Ku80 heterodimer or Ku70 homodimer, which after binding to DNA free ends undergo allosteric change, thereby providing a scaffold for recruitment of DNA-PKcs. The protein kinase activity of Ku/DNA-PKcs complex recruits accessory factors to modify free DNA ends, which cannot be ligated directly (Davis et al., 2014). Then, XRCC4 and XLF also assemble at DSB sites, aligns the chromatin in the vicinity, and mediates recruitment of Ligase IV which carry out the final (ligation) step of the repair. These core components are sufficient to recognize DSBs, align the broken DNA fragments, and anneal them, fixing the DSBs (Chang et al., 2017).

*PAXX* (Paralog of *XRCC4* and XLF; previously called C9orf142) is a recently characterized protein associated with the classical NHEJ pathway. It structurally resembles XRCC4 and XLF and facilitates the assembly of the core NHEJ complex at the DNA damage site (Ochi et al., 2015; Kumar et al., 2016). Although, PAXX and XLF perform overlapping functions and XLF can efficiently compensate for PAXX deficiency in colon cancer cells (Tadi et al., 2016), however, another study demonstrated that one protein between PAXX and XLF is essential for NHEJ repair, and PAXX also promotes Ku accumulation at DSBs (Liu et al., 2017). Interestingly, a recent study reported the synergistic role of PAXX, XRCC4, and XLF in the recruitment of DNA Pol λ as an accessory factor for DNA damage repair (Craxton et al., 2018). Although, these studies suggest that both PAXX and XLF perform overlapping but essential functions in NHEJ mediated DNA repair and influence drug resistance in solid tumors, the consequences and clinical implications of their altered expression in cancer patients have never been investigated. While XLF confers resistance to oxaliplatin and 5-fluorouracil in CRC cells (Liu et al., 2019), PAXX overexpression is associated with drug resistance in osteosarcoma cells (Ma et al., 2020).

Non-homologous end joining pathway genes in this CRC harbor both genetic and epigenetic alterations which promote cancer progression (Beggs et al., 2012; Mijnes et al., 2018). Variations at the 3′UTR of mRNA encoding DSB repair proteins have also been associated with a higher risk of CRC and poor outcome of the disease (Naccarati et al., 2015). Conventional cancer therapies including radiation and chemotherapy primarily exert their effect by inducing DSBs mediated cancer cell death. Therefore, the NHEJ pathway genes are considered as potential therapeutic targets to overcome drug resistance in CRC. Previous reports have analyzed the expression of NHEJ genes in different cancers, including some in colorectal cancer (Sishc and Davis, 2017). In the present study, we performed a comprehensive analysis of the core NHEJ pathway genes using well characterized multi-omics datasets to determine the deregulated expression pattern and clinical significance of NHEJ pathway genes in colon cancer.

## Materials and Methods

### Data Acquisition and Analysis

Oncomine^1^ a web online database was used to analyze the expression of mRNA encoded by NHEJ genes, in several colon cancer datasets. The parameters for comparing gene expression between normal and tumor tissues included mRNA data with a threshold of *p* < 0.01 with any fold change.

Gene expression and DNA methylation of colon cancer developed by The Cancer Genome Atlas (TCGA-COAD study) was extracted as fragment per kilobase million (FPKM) values from the UCSC Xena browser,^2^ and used for subsequent analysis. Similarly, information about clinical features and tumor mutation status of colon cancer patients of TCGA study was retrieved from cBioportal^3^ by selecting the TCGA PanCancer Atlas - Colorectal Adenocarcinoma study and selecting patients with colon adenocarcinoma in cancer type (Cerami et al., 2012; Gao et al., 2013).

High throughput total protein and phosphoprotein estimation data for 100 normal colon and 97 colon cancer tissues, generated by mass spectrometry (MS) in Clinical Proteomic Tumor Analysis Consortium (CPTAC) study was analyzed using UALCAN web server^4^ (Chen et al., 2019). The *z*-value, used to compare protein levels (depicted on the *y*-axis) represents the standard deviation from the median across samples. As described in UALCAN web server, log2 spectral count ratio values, downloaded from CPTAC colon cancer data were normalized within each sample profile and then normalized across samples to calculate *z*-values as relative protein levels. Available total protein and phosphoprotein levels of the NHEJ pathway were assessed using default parameters in the UALCAN web server.

MEXPRESS web server^5^ hosts the DNA methylation data from TCGA studies developed on “Illumina Human Methylation 450 Bead Chip” platform and provides access to methylation levels of designated CpG sites of the queried gene and its association with gene expression (Koch et al., 2015). For DNA methylation analysis, correlation of PAXX expression with the methylation status of its gene was determined using the MEXPRESS web server using default parameters.

### Survival Analysis

Kaplan Meier plot was constructed along with log-rank test *p*-values using the “survminer” package in R statistical software (version 4.0.1). Briefly, patients were categorized into high and low expression groups based on median gene expression values in FPKM (extracted from UCSC Xena browser). Univariate analysis was performed for overall survival (OS), disease-specific survival (DSS), disease-free interval (DFI), and progression-free interval (PFI) to establish the association of gene expression and clinicopathological parameters with patient outcome. Multivariate analysis was also performed for genes, which were significantly associated with prognosis in univariate analysis. Important clinical and molecular features, including age, gender, stage, histological subtype, KRAS status, and BRAF status were taken as covariates.

### Pathway Analysis

Gene expression correlations of *PAXX* with whole gene expression profiles of colon cancer tissues from TCGA-COAD dataset were extracted from the cBioPortal web server (see text footnote 3). Briefly, *PAXX* expression was used as input in colon adenocarcinoma patient data from TCGA colon cancer (TCGA-COAD PanCancer study) dataset (Cerami et al., 2012; Gao et al., 2013) in cBioPortal. Then, by using the correlation module, the whole transcriptome correlations table of *PAXX* expression was retrieved. After filtering correlations with false detection rate normalized *q*-value < 0.05, genes were arranged by increasing value of Spearman’s correlation constant, thus creating a ranked gene file. The ranked gene file was further used as input for the pre-ranked GSEA module in the gene set enrichment analysis tool from Broad Institute^6^ with predefined molecular signature database hallmark gene set (version 7.1) as reference gene set for pathway enrichment (Liberzon et al., 2015). Genes enriched in the respective pathways were represented as direct image outputs along with calculated normalized enrichment score (NES), false discovery rate (FDR), and *p*-value.

### Protein Interaction Analysis

Biophysical interactions of ORFeome-based complexes (BioPlex) network interactome tool,^7^ a large-scale interactome database based on affinity purification mass spectrometry (AP-MS) data of baits from the human ORFeome (Huttlin et al., 2017) was utilized to identify PAXX interacting proteins in colon cancer cells HCT-116. Then an interaction network of PAXX associated proteins in these cells was constructed using default parameters.

### TISIDB Analysis

The tumor-immune interactions database (TISIDB)^8^ is an integral web portal for the interaction of tumor and immune system (Ru et al., 2019). This database enabled us to correlate *PAXX* gene expression and infiltration of different immune cells types including CD8 T cells (activated, central memory and effector memory), CD4 T cells (activated, central memory and effector memory), T helper cells (follicular, type 1 and 2), gamma delta T cells, B-cells (activated, immature and mature), dendritic cells (activated, plasmacytoid and immature), NK cells, macrophages, eosinophil, mast cell, neutrophils, and monocytes. Immune cell fractions were determined using the computational “deconvolution” approach, which is based on determining mRNA contribution from immune cells from the bulk tumor RNA-sequencing profile.

### Statistical Analysis

Gene expression analyses were performed on Graphpad Prism (version 6). Mann-Whitney *U*-test was used for comparing gene expression between normal and colon cancer tissues. *P*-value < 0.05 was considered statistically significant. Wilcoxon paired *t*-test was applied for paired expression analysis between normal and colon cancer tissues. Level of significance denoted on the expression graphs were represented as ^∗^*p*-value < 0.05, ^∗∗^*p*-value < 0.01, ^∗∗∗^*p*-value < 0.001 and ^****^p-value < 0.0001. Patients were divided into two groups by median expression and a log-rank test was used to compare groups for Kaplan-Meier survival analysis. Univariate and multivariate survival data analysis were performed on Stata version 11.

## Results

### mRNA Expression Pattern of NHEJ Pathway Genes in Colon Cancer

To determine the expression pattern of core NHEJ genes in colon cancer, we performed Oncomine analysis for *XRCC6* (Ku70), *XRCC5* (Ku80), *PRKDC* (DNA-PKcs), *XRCC4* (XRCC4), *LIG4* (DNA ligase 4), *NHEJ1* (XLF), and *PAXX* (PAXX/XLS). It provided the advantage of analyzing several datasets in parallel to assess the general expression pattern of these genes. This analysis revealed significant upregulation of five genes, (*XRCC6*, *XRCC4*, *PRKDC*, *XRCC4*, and *PAXX*) and downregulation of two (*LIG4* and *NHEJ1)* NHEJ pathway genes, in tumor tissues compared to the normal tissues (Table 1).

**TABLE 1:** Expression of NHEJ pathway genes in colon cancer determined by Oncomine analysis.

	Upregulated		Downregulated	
		
Gene Name	Analysis meet threshold of *p* < 0.001	Datasets covered in the analysis that meet the threshold	Analysis meet the threshold of *p* < 0.001	Datasets covered in the analysis that meet the threshold
*XRCC6*	9/25	5/10	0/25	0/10
*XRCC5*	17/27	9/12	0/27	0/12
*PRKDC*	22/26	10/11	0/26	0/11
*XRCC4*	11/25	7/10	0/25	0/10
*LIG4*	0/25	0/10	8/25	5/10
*NHEJ1*	1/23	1/8	13/23	6/8
*PAXX*	10/24	5/9	0/24	0/9

**FIGURE 1:**
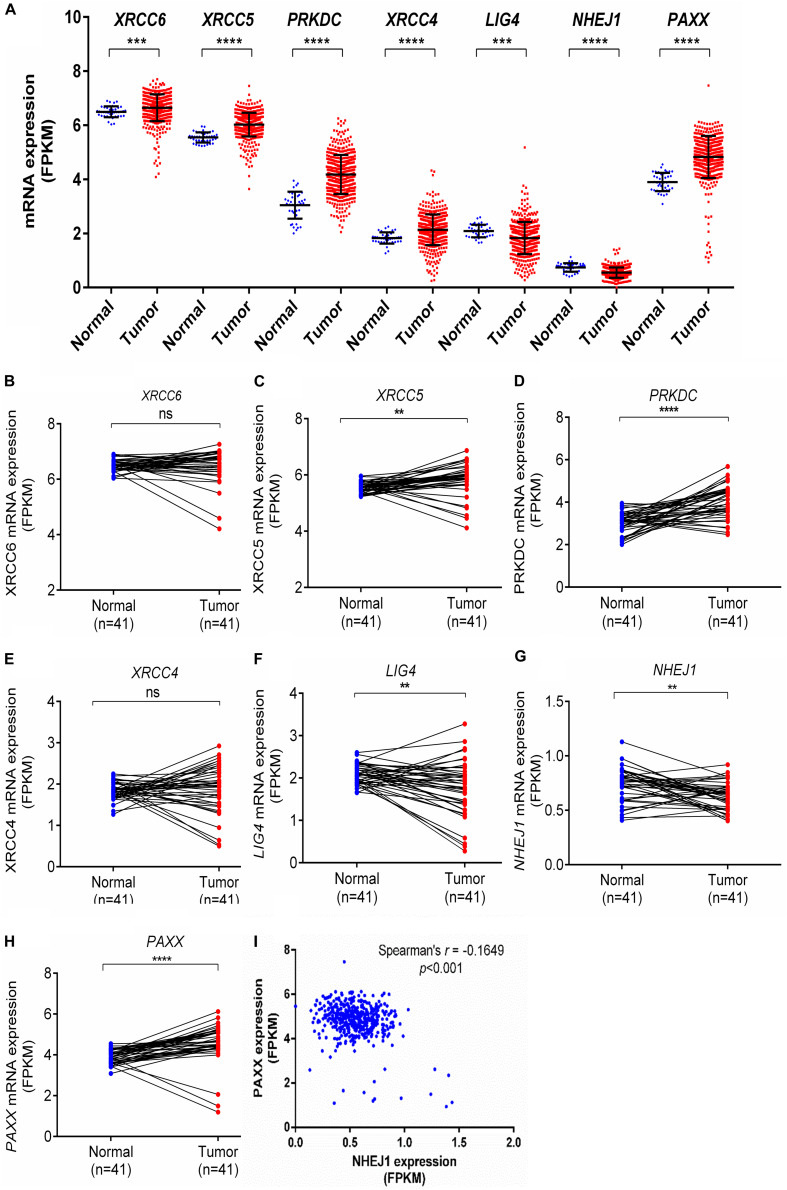
Comparison of mRNA expression of NHEJ pathway genes in normal colon and colon cancer tissues from TCGA-COAD dataset including total samples for seven NHEJ genes **(A)**, and paired samples, including *XRCC6* **(B)**, *XRCC5* **(C)**, *PRKDC* **(D)**, *XRCC4* **(E)**, *LIG4* **(F)**, *NHEJ1* **(G)**, and PAXX **(H)**. *p < 0.05, **p < 0.01, ***p < 0.001, ****p < 0.0001. **(I)** Correlation between *NHEJ1* and *PAXX* expression in the same dataset. FPKM, Fragments per kilo million bases.

To corroborate our findings, we utilized a dataset of colon cancer from The Cancer Genome Atlas (TCGA) to compare the expression of NHEJ pathway genes between tumors and normal colon tissues. Consistent with the Oncomine analysis, comparison of all available normal (*n* = 41) and tumor tissues (*n* = 469) revealed overexpression of *XRCC6*, *XRCC5*, *PRKDC*, *XRCC4*, and *PAXX* in tumors compared to normal tissues, while *LIG4* and *NHEJ1* displayed lower expression in the tumor tissues (Figure 1A). However, analysis of 41 paired normal and tumor tissues revealed significant overexpression of only *XRCC5*, *PRKDC*, and *PAXX* genes in tumor tissues compared to the normal colon (Figures 1C,D,H, respectively), while *LIG4* and *NHEJ1* still displayed reduced expression (Figures 1F,G, respectively). Interestingly, in contrast to Oncomine analysis, *XRCC6* and *XRCC4* did not display differential expression between paired normal and tumor tissues (Figures 1B,E, respectively).

Co-expression analysis among all NHEJ pathway genes in TCGA-COAD dataset revealed a negative correlation between *PAXX* and *NHEJ1* expression (Figure 1I). While the expression of all other genes of this pathway exhibited positive correlations among them (Supplementary Table S1).

### Expression of Proteins Encoded by NHEJ Pathway Genes in Colon Cancer

**FIGURE 2:**
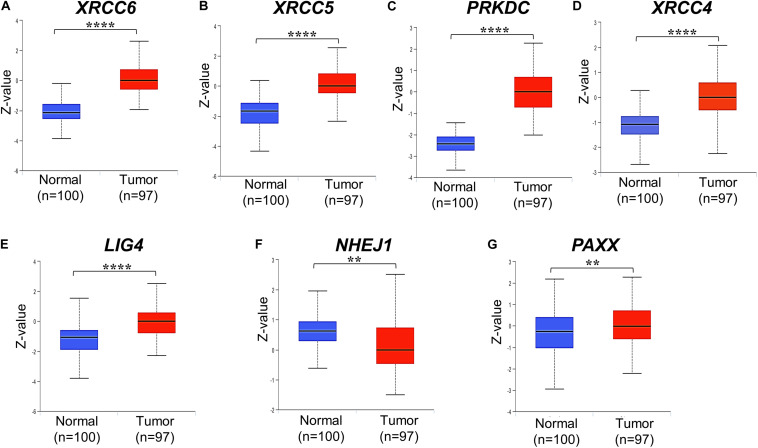
Total protein levels of NHEJ pathway genes in normal tissues and colon cancer tissues from CPTAC study, including **(A)** XRCC6 **(B)** XRCC5 **(C)** PRKDC **(D)** XRCC4 **(E)** LIG4 **(F)** NHEJ1 and **(G)** PAXX. *p < 0.05, **p < 0.01, ***p < 0.001, ****p < 0.0001.

Further, the Clinical Proteomic Tumor Analysis Consortium (CPTAC) dataset, which consists of high throughput mass spectrometry based quantitative protein estimation data of colon cancer and respective normal colon tissues, was used to compare total and phosphorylated protein levels of NHEJ pathway in normal colon tissues (*n* = 100) and colon cancer (*n* = 97). Consistent with the Oncomine gene expression analysis, total protein levels of Ku70 (*XRCC6*), Ku80 (*XRCC5*), DNA-PKcs (*PRKDC*), XRCC4, and PAXX were found to be significantly higher in colon cancer tissue compared to normal colon tissues, while *LIG4*, which exhibited reduced mRNA expression in Oncomine analysis, also displayed higher total protein levels in tumors (*p* < 0.01 for all, Figures 2A–G). However, NHEJ1 protein levels in line with the Oncomine analysis were observed to be lower in tumor tissues compared to the controls (*p* < 0.001, Figure 2F).

DNA-PKcs has been shown to phosphorylate many of the core NHEJ factors *in vitro*, but most of these phosphorylations are non-essential for NHEJ function (Davis et al., 2014). We observed that some uncharacterized phosphorylated protein levels of Ku70 (*XRCC6*, position Ser520, and Thr455), DNA-PKcs (*PRKDC*, Ser893, Ser3995 and Ser3205), and PAXX (Ser148) were higher in colon cancer tissues compared to normal colon tissues (Supplementary Figures S1A–K), whereas phosphorylated XLF (*NHEJ1*, Ser287) was lesser in colon cancer tissues.

Interestingly, it has been previously demonstrated that PRKDC is phosphorylated at Ser3995 in response to IR radiation, by ATM serine/threonine kinase (ATM) protein, but this phosphorylation does not affect NHEJ repair (Neal et al., 2011). Further, Douglas et al. (2014) reported that DNA-PKcs is phosphorylated and dephosphorylated at Ser3205 by PLK1 (polo-like kinase 1) and PP6 (protein phosphatase 6), respectively during mitosis. Phospho-mimicry of PAXX phosphorylation at Ser134, Thr145, Ser148, and Ser152 has been reported to destabilize the PAXX-Ku-DNA ternary complex, but it does not affect the stimulation of LIG4/XRCC4 blunt-ended DNA-ligation activity by PAXX (Tadi et al., 2016). Therefore, the exact role of the modifications of DNA-PKcs and PAXX in NHEJ activity remains unclear and warrants further studies.

### Associations of NHEJ Pathway Gene Expression With Clinicopathological Features in Colon Cancer

**FIGURE 3:**
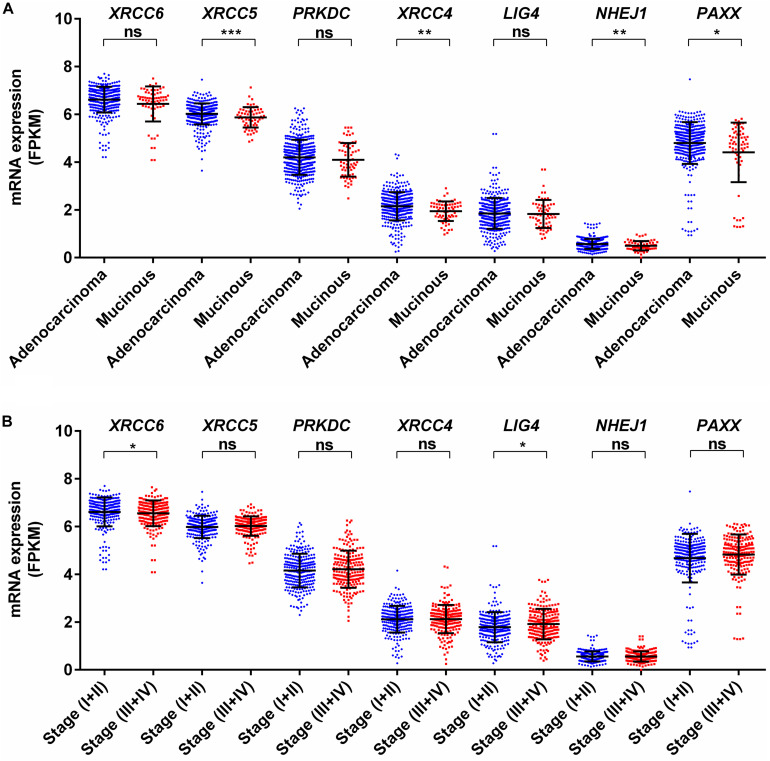
Association of mRNA expression of NHEJ pathway genes with **(A)** tumor histology, and **(B)** stage. *p < 0.05, **p < 0.01, ***p < 0.001, ****p < 0.0001. FPKM, Fragments per kilo million bases.

We analyzed associations of NHEJ pathway gene expression with other clinicopathological features, such as age, gender, histological type, stage, *KRAS* mutation status, *BRAF* mutation status, and microsatellite instability (MSI) status in colon cancer by performing a direct comparison between mRNA expressions of respective genes. None of the seven NHEJ pathway genes analyzed in the present study displayed any association with age (Supplementary Figures S2A–G). Only *LIG4* was associated with gender and exhibited higher expression in males compared to females (Supplementary Figure S3E). Between two histological subtypes, no difference was observed in the expression of *XRCC6*, *PRKDC*, *LIG4*, and *PAXX*, while expression of *XRCC5*, *XRCC4*, and *NHEJ1* was higher in adenocarcinoma compared to mucinous adenocarcinoma (Figure 3A). We further compared the mRNA expression of NHEJ genes between stage (I + II) group with stage (III + IV) group colon tumors. This analysis revealed reduced expression of *XRCC6* in advanced stage group while *LIG4* displayed elevated expression in the same group (Figure 3B). However, no difference in mRNA levels of *XRCC5*, *PRKDC*, *XRCC4*, *NHEJ1*, and *PAXX* between the two groups.

**FIGURE 4:**
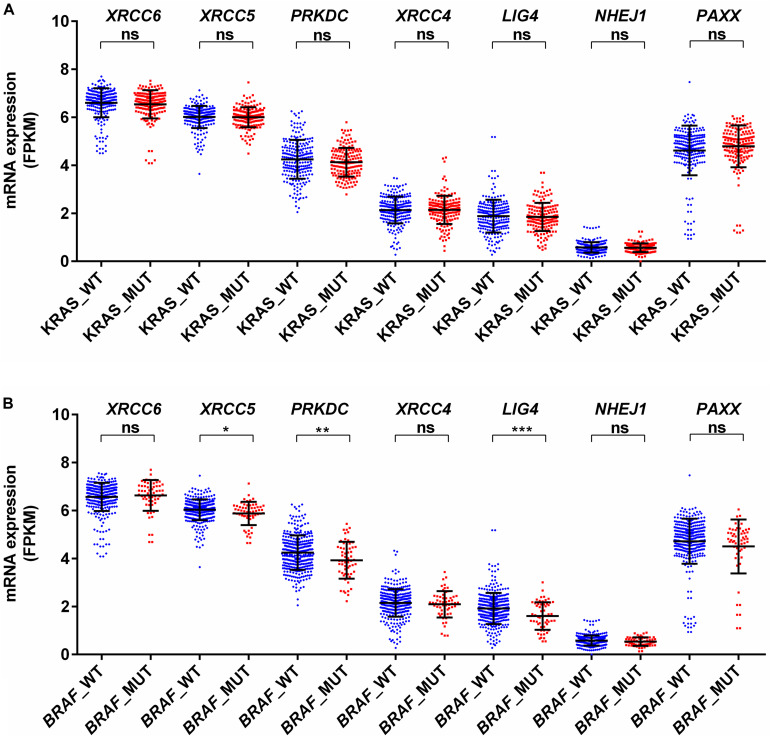
Association of mRNA expression of NHEJ pathway genes with **(A)** *KRAS* mutation status, and **(B)** *BRAF* mutation status. *p < 0.05, **p < 0.01, ***p < 0.001, ****p < 0.0001. FPKM, Fragments per kilo million bases. WT, wild type; MUT, mutant.

*KRAS* mutations have been reported to enhance homologous recombination repair in preference to NHEJ in colorectal cancer cells (Kalimutho et al., 2017). In agreement with this report, we observed no difference for mRNA expression in all analyzed genes between KRAS wild type and mutant tumors (Figure 4A). Therefore, *KRAS* mediated oncogenic reprogramming does not seem to be involved in the altered NHEJ pathway in colon cancer. Concerning *BRAF* mutation in thyroid cancer, two reports have demonstrated that *BRAF* mutation promotes NHEJ activity through upregulation of *NHEJ1* and it is also associated with radioresistance (Robb et al., 2018, 2019). In a melanoma cell line model, it has been shown that mutant *BRAF* inhibition may increase DNA damage by downregulation of NHEJ pathway genes, including *XRCC6*, *XRCC5*, and *PRKDC* (Fatkhutdinov et al., 2016). Our analysis revealed that *BRAF* mutant colon cancer did not harbor higher *NHEJ1* expression compared to *BRAF* wild type tumors and three NHEJ pathway genes, *XRCC5*, *PRKDC*, and *LIG4* are indeed lowly expressed in *BRAF* mutant tumors (Figure 4B). These results suggest that a detailed study of the NHEJ pathway concerning *BRAF* mutation in colon cancer is further warranted.

**FIGURE 5:**
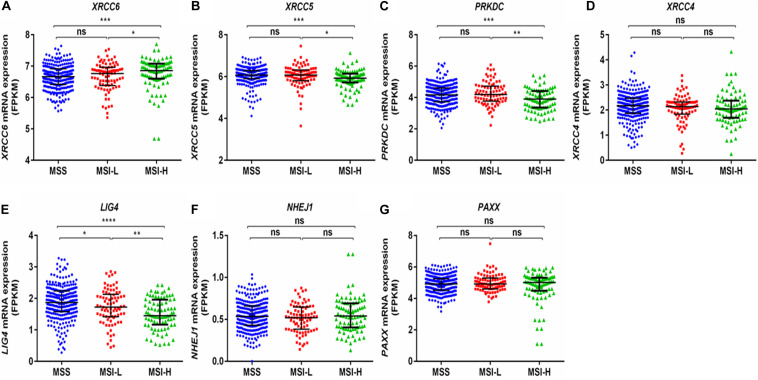
Comparison of mRNA expression of NHEJ pathway genes based on microsatellite instability status from TCGA-COAD dataset. **(A)** XRCC6 **(B)** XRCC5 **(C)** PRKDC **(D)** XRCC4 **(E)** LIG4 **(F)** NHEJ1, and **(G)** PAXX. FPKM, Fragments per kilo million bases. MSI-H, MSI-high; MSI-L, MSI-low; MSS, microsatellite stable. *p < 0.05, **p < 0.01, ***p < 0.001, ****p < 0.0001.

Interestingly, a previous report suggests that the NHEJ pathway is impaired in several mismatch repair deficient colon cancer cell lines (Koh et al., 2005). We observed that expression of *XRCC6* was higher in MSI-high tumors compared to MSI-low and microsatellite stable (MSS) tumors, while *XRCC5*, *PRKDC*, and *LIG4* exhibited reduced expression in MSI-high tumors compared to both MSI-low and MSS tumors (Figures 5A–G).

### Survival Analysis

**FIGURE 6:**
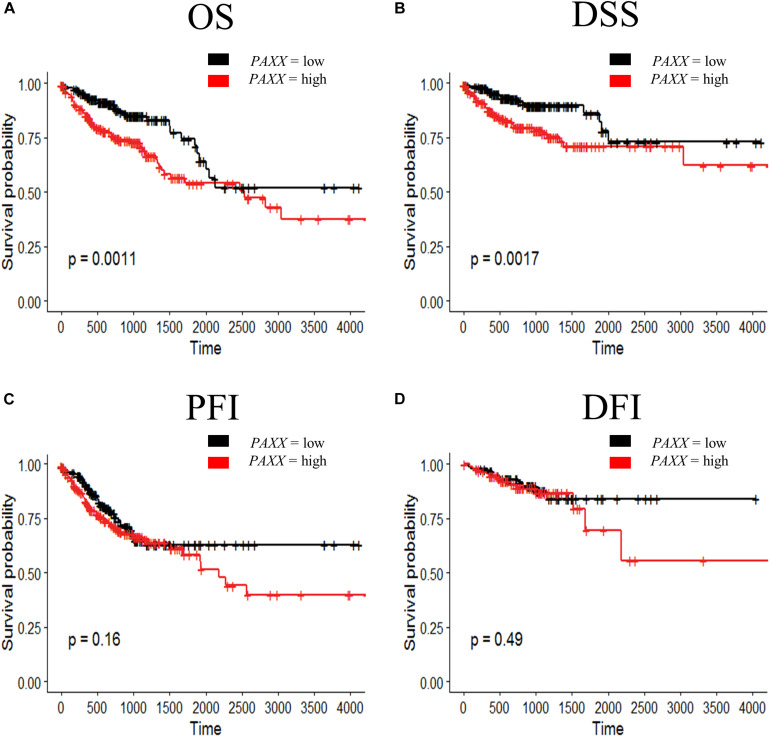
Kaplan-Meier survival curve for prognostic significance of PAXX gene expression in TCGA-COAD dataset, including **(A)** overall survival (OS), **(B)** disease-specific survival (DSS), **(C)** progression-free interval (PFI), and **(D)** disease-free interval (DFI). Survival probabilities are presented on the *y*-axis and time in days on the *x*-axis in all graphs. The log-rank test *p*-value has been depicted in respective graphs.

To further determine the clinical significance of the expression of NHEJ pathway genes in colon cancer, we performed Kaplan-Meier survival analysis for overall survival (OS), disease-specific survival (DSS), progression-free interval (PFI), and disease-free interval (DFI) using TCGA colon cancer dataset. We observed that among all NHEJ pathway genes only elevated *PAXX* expression was associated with poor overall survival (*p* = 0.0011, Figure 6A), while other genes did not display significant association with OS (Supplementary Figure S4), DSS (Supplementary Figure S5), or PFI (Supplementary Figure S6). Interestingly, *PAXX* overexpression was also associated with poor DSS (*p* = 0.0011, Figure 6B), but not with PFI or DFI (Figures 6C,D, respectively). Furthermore, higher *XRCC4* expression was associated with poor DFI (Supplementary Figure S7D).

To assess the robustness of these gene products as prognostic biomarkers, we performed univariate analysis followed by a multivariate analysis using a Cox proportional hazards model. Gene expression was taken as a continuous variable while important clinical features including age, gender, stage, *KRAS* mutation, *BRAF* mutation, and MSI status were taken as covariates. The results of univariate analysis have been presented in Table 2. Interestingly, we observed that only *PAXX* overexpression was associated with poor OS and DSS, while the levels of other gene products were not associated with OS, DSS, PFI, or DFI. Therefore, the expression of *PAXX* was considered for multivariate analysis. Interestingly, in multivariate analysis, *PAXX* overexpression emerged as an independent marker for poor OS and DSS (Table 3).

**TABLE 2:** Univariate analysis of the NHEJ pathway genes and clinicopathological parameters in TCGA-COAD dataset.

	OS			DSS			DFI			PFI		
				
Variable	HR	95% CI	*p*-value	HR	95% CI	*p*-value	HR	95% CI	*p*-value	HR	95% CI	*p*-value
Age	1.017	1.000–1.033	0.041	0.996	0.977–1.016	0.745	1.015	0.981–1.051	0.372	0.996	0.982–1.011	0.675

**Gender**	**Ref**			**Ref**			**Ref**			**Ref**		

Male										0.811	0.564–1.166	0.260
Female	0.898	0.603–1.335	0.596	0.901	0.540–1.500	0.689	0.592	0.251–1.396	0.231			

**Stage I**	**Ref**			**Ref**			**Ref**			**Ref**		

II	2.341	0.819–6.694	0.112	3.294	0.416–26.040	0.258	2.020	0.567–7.195	0.278	2.327	0.979–5.530	0.056
III	4.648	1.650–13.094	0.004	10.45945	1.398–78.212	0.022	2.043	0.539–7.734	0.293	3.707	1.561–8.803	0.003
IV	11.573	4.080–32.827	0.000	.001	6.132–330.249	0.000				14.456	6.113–34.181	0.000

**Histology: COAD**	**Ref**			**Ref**			**Ref**			**Ref**		

Mucinous COAD	1.300	0.759–2.225	0.338	0.991	0.470–2.088	0.983	0.328	0.044–2.441	0.277	1.029	0.608–1.744	0.913

**MSI status: MSS**	**Ref**			**Ref**			**Ref**			**Ref**		

MSI-L	1.205	0.732–1.984	0.461	1.282	0.692–2.372	0.429	1.370	0.530–3.539	0.515	1.456	0.939–2.258	0.093
MSI-H	0.918	0.534–1.577	0.758	0.791	0.381–1.642	0.531	0.320	0.072–1.407	0.132	0.828	0.493–1.390	0.476

***KRAS*: WT**	**Ref**			**Ref**			**Ref**			**Ref**		

Mutation	0.982	0.642–1.501	0.933	1.519	0.874–2.641	0.138	2.240	0.927–5.408	0.073	1.770	1.203–2.604	0.004

***BRAF*: WT**	**Ref**			**Ref**			**Ref**			**Ref**		

Mutation	1.159	0.663–2.026	0.604	0.613	0.243–1.547	0.301	0.661	0.153–2.845	0.579	0.793	0.443–1.417	0.434

**Gene expression**												

*PAXX* expression	**1.560**	**1.164–1.164**	0.003*	**1.908**	**1.280**–**2.844**	0.002*	1.315	0.796–2.172	0.283	1.153	0.942–1.411	0.166
*NHEJ1* expression	0.774	0.288–2.080	0.613	0.298	0.076–1.166	0.082	0.869	0.144–5.221	0.878	0.718	0.305–1.690	0.449
*XRCC4* expression	1.051	0.723–1.527	0.794	1.164	0.723–1.872	0.531	1.816	0.880–3.749	0.106	1.042	0.747–1.454	0.806
*XRCC5* expression	0.922	0.587–1.450	0.728	1.067	0.591–1.926	0.829	1.768	0.747–4.182	0.195	1.055	0.701–1.589	0.795
*XRCC6* expression	1.075	0.762–1.517	0.679	1.127	0.724–1.754	0.594	1.433	0.641–3.204	0.380	1.000	0.749–1.335	0.998
*PRKDC* expression	0.860	0.652–1.135	0.289	0.837	0.588–1.192	0.325	0.864	0.491–1.520	0.614	0.954	0.744–1.225	0.717
*LIG4* expression	1.010	0.745–1.368	0.947	0.978	0.663–1.445	0.915	1.242	0.742–2.080	0.408	1.060	0.813–1.381	0.665

OS, overall survival; DSS, disease-specific survival; DFI, disease-free interval; PFI, progression-free interval; HR, hazard ratio; CI, confidence interval; * indicates *p* < 0.05. Bold values represent any significant association of analyzed gene with patient prognosis.

**TABLE 3:** Multivariate analysis of *PAXX* expression and clinicopathological parameters in TCGA-COAD dataset.

	OS			DSS			DFI			PFI		
Variable	HR	95% CI	*p*-value	HR	95% CI	*p*-value	HR	95% CI	*p*-value	HR	95% CI	*p*-value
Age	1.038	1.018–1.058	0.000	1.022	0.998–1.047	0.065	1.002	0.959–1.048	0.903	1.006	0.989–1.023	0.436

**Gender**	**Ref**			**Ref**			**Ref**			**Ref**		

Male												
Female	1.049	0.658–1.673	0.839	1.263	0.708–2.252	0.428	0.526	0.190–1.460	0.218	0.849	0.561–1.287	0.442

**Stage I**	**Ref**			**Ref**			**Ref**			**Ref**		

II	2.614	0.784–8.718	0.118	2.640	0.327–21.280	0.362	2.274	0.622–8.300	0.214	3.265	1.152–9.249	0.026
III	6.039	1.809–20.155	0.003	10.240	1.346–77.872	0.025	2.257	0.542–9.398	0.263	6.349	2.227–18.101	0.001
IV	20.672	6.042–70.719	0.000	52.407	6.937–395.919	0.000				21.135	7.284–61.319	0.000

**Histology: COAD**	**Ref**			**Ref**			**Ref**			**Ref**		

Mucinous COAD	1.495	0.820–2.725	0.189	0.931	0.387–2.238	0.874	0.00	0.000–0.000	1.000	0.870	0.460–1.646	0.670

**MSI status: MSS**	**Ref**			**Ref**			**Ref**			**Ref**		

MSI-L	1.305	0.750–2.269	0.346	1.417	0.726–2.766	0.306	1.457	0.536–3.958	0.460	1.799	1.127–2.873	0.014
MSI-H	0.967	0.403–2.318	0.941	1.376	0.430–4.404	0.590	0.268	0.033–2.144	0.215	1.462	0.709–3.017	0.303

***KRAS*: WT**	**Ref**			**Ref**			**Ref**			**Ref**		

Mutation	1.178	0.714–1.944	0.520	1.955	1.062–3.601	0.031	2.062	0.804–5.287	0.132	1.918	1.247–2.949	0.003

***BRAF*: WT**	**Ref**			**Ref**			**Ref**			**Ref**		

Mutation	1.694	0.691–4.153	0.249	1.337	0.362–4.940	0.663	2.842	0.356–22.656	0.324	1.251	0.539–2.903	0.601

***PAXX* expression**	**1.562**	**1.065**–**2.291**	0.022*	**1.858**	**1.122**–**3.075**	0.016*	1.036	0.495–2.167	0.924	1.049	0..764–1.442	0.764

OS, overall survival; DSS, disease-specific survival; DFI, disease-free interval; PFI, progression-free interval; HR, hazard ratio; CI, confidence interval; * indicates *p* < 0.05. Bold values represent any significant association of analyzed gene with patient prognosis.

### Methylation Analysis of the *PAXX* Gene

**FIGURE 7:**
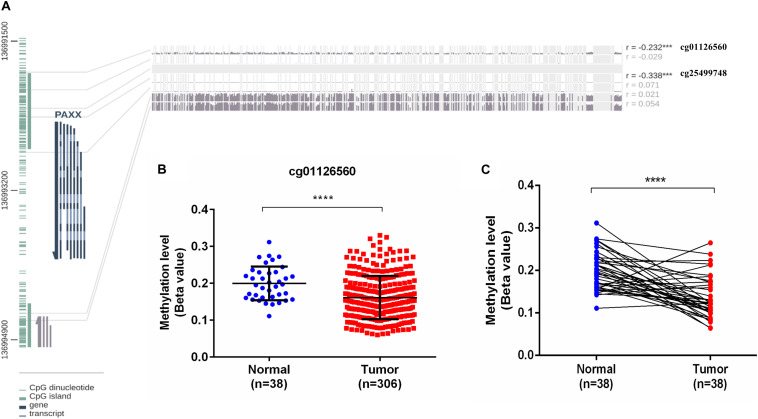
DNA methylation analysis of the PAXX gene from TCGA-COAD dataset. **(A)** Graphical representation of CpG Island and sites around PAXX genomic locus with Pearson’s correlation values of PAXX expression with methylation of different CpG sites. **(B)** Comparison of DNA methylation levels of cg01126560 between total normal and colon cancer tissues. **(C)** Comparison of DNA methylation levels of cg01126560 between paired normal colon and colon cancer tissues. *p < 0.05, **p < 0.01, ***p < 0.001, ****p < 0.0001.

*PAXX* gene contains a CpG island spanning its transcription start site (TSS) and the first two exons (Figure 7A). Given this information, it was of interest to investigate the role of epigenetic modifications in the overexpression of *PAXX* in colon tumors. For this purpose, we assessed DNA methylation and paired RNA expression data of TCGA-COAD through the MEXPRESS web server. Pearson correlation analysis between methylation of five CpG sites of *PAXX* promoter and transcription of its gene revealed that DNA methylation of two distinct sites captured by probes, cg01126560 and cg25499748 exhibited significant negative correlation to *PAXX* gene expression in TCGA-COAD dataset (*r* = −0.232, *p* < 0.001 and *r* = −0.338, *p* < 0.001, respectively, Figure 7A). Further, the level of methylation of cg01126560 was lower in a group of all available colon cancer tissues compared to normal tissues (*p* < 0.0001, Figure 7B). Furthermore, a comparison of paired colon cancer tissues with respective normal tissues also revealed that colon cancer tissues exhibit lower methylation of cg01126560 (*p* < 0.0001, Figure 7C). These results suggested the involvement of methylation in transcriptional regulation of *PAXX* expression in colon carcinoma.

### Cellular Pathways Associated With *PAXX* Expression in Colon Cancer

**FIGURE 8:**
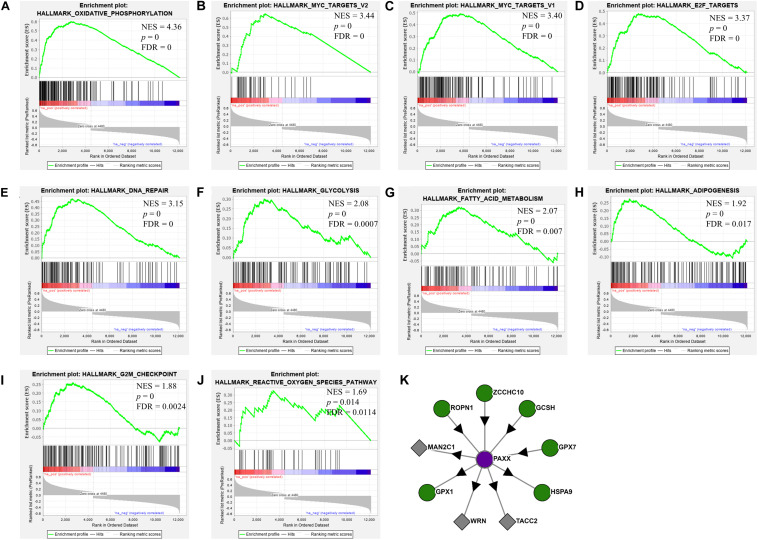
Gene set enrichment analysis of PAXX correlated genes in the TCGA-COAD dataset. Each plot **(A–J)** depicts positively enriched pathways of PAXX correlated genes with normalized enrichment score (NES), false discovery rate (FDR), and *p*-value depicted inside the respective pathway. **(K)** Depict results from the Bioplex 2.0 web server, showing protein-protein interactions of PAXX in HCT-116 colon cancer cell line.

To assess the oncogenic pathways associated with *PAXX* expression in colon cancer, we performed gene set enrichment analysis (GSEA) for cancer hallmarks pathways using genes that exhibited significant correlations with *PAXX*. Among positively correlated pathways, *PAXX* expression exhibited the most significant correlation with oxidative phosphorylation (Figure 8A), besides other metabolic pathways including glycolysis (Figure 8F), fatty acid metabolism (Figure 8G), and adipogenesis (Figure 8H). We also observed a positive correlation of PAXX with DNA repair (Figure 8E), MYC targets (Figures 8B,C), E2F targets (Figure 8D), G2M checkpoint (Figure 8I), and reactive oxygen species (Figure 8J), pathways. Further, protein interaction data of *PAXX* protein in HCT-116 colon cancer cell line from “Bioplex 2.0” database also revealed interaction of *PAXX* with Werner syndrome ATP-dependent helicase (*WRN*), an established mediator of NHEJ pathway, supporting the involvement of *PAXX* in NHEJ pathway in colon cancer (Figure 8K). Interestingly, PAXX was also observed to interact with genes involved in glutathione metabolism, including glutathione peroxidase 1 and 7 (GPX1 and GPX7, respectively), which are primarily involved in protecting cells from oxidative stress, suggesting additional pro-tumor roles of PAXX in conferring therapeutic resistance to colon cancer cells.

We observed a significant negative correlation between PAXX expression and epithelial to mesenchymal transition pathway (Supplementary Figure S8A). Other pathways that exhibited a negative correlation with PAXX included downregulated genes in UV response, KRAS signaling, Hedgehog signaling, and angiogenesis (Supplementary Figures S8B–E). Interestingly, we also observed a negative correlation of PAXX with immunity associated pathways including inflammatory response, TGF beta signaling, and complement pathway (Supplementary Figures S8F–H). We further correlated *PAXX* expression with the computationally determined abundance of different tumor-infiltrating immune cells in TCGA-COAD dataset. *PAXX* was observed to be negatively correlated with twenty different immune cells, thereby suggesting the association of *PAXX* expression with overall reduced tumor immune infiltration in colon cancer (Supplementary Figure S9).

## Discussion

Aberrations in the NHEJ pathway are common in cancers. Hosoi et al. reported elevated expression of Ku70 and Ku80 mRNA as well as proteins in colorectal carcinoma compared to the normal colon (Hosoi et al., 2004). In contrast, Beggs et al. reported reduced expression of Ku70 in colon cancer cells, which was associated with higher genomic instability (Beggs et al., 2012). In another study, it was observed that cytoplasmic Ku70 protein levels are higher in patients who do not respond to chemoradiotherapy, while Ku80 was lost in those patients (Pucci et al., 2017). Thus, previous studies have described both overexpression and downregulation of NHEJ pathway genes in colorectal cancer. Also, some of these studies have estimated mRNA levels while others have assessed protein expression. To resolve this paradox, we performed a comprehensive analysis of the core NHEJ pathway genes in colon cancer. Our analysis revealed elevated mRNA and protein expression of *XRCC6* (Ku70) and *XRCC5* (Ku80) in colon cancer compared to normal colon tissue. Furthermore, the overexpression pattern is more robust for *XRCC5* as observed in paired normal and tumor tissue comparison, while *XRCC6* did not exhibit significant difference. Indeed, we observed reduced *XRCC6* expression in tumors at an advanced stage (stage III + IV) compared to the lower stage (stage I + II).

*PRKDC* exhibited overexpression in Oncomine analysis, TCGA dataset as well as CPTAC study suggesting consistent overexpression of this protein in colon cancer, both at the mRNA and protein levels. *PRKDC* expression was not associated with age, gender, stage, and histology. A previous study had also reported higher mRNA and protein levels of *PRKDC* in colorectal cancer tissues compared to normal tissues, which also exhibited a positive correlation with expression of *XRCC6* and *XRCC5* (Hosoi et al., 2004). In our analysis these three proteins exhibited a significant positive correlation with each other. Further, a recent report highlighted the dependency of colorectal cancer cells on *PRKDC* and also showed that *PRKDC* overexpression in colon cancer is associated with poor OS (Sun et al., 2016). While we observed a similar pattern of overexpression of *PRKDC* in colon cancer, its mRNA expression was not associated with any of the four types of survival parameters analyzed. Therefore, the collective data along with our results validate *PRKDC* overexpression as a potential therapeutic target in colon cancer.

Gene polymorphism in *XRCC4* has been associated with CRC risk (Bau et al., 2010; Zhang and Hu, 2011). Our analysis revealed elevated levels of *XRCC4* mRNA in Oncomine and protein data analysis, respectively, whereas the comparison of expression between paired normal and tumor tissues in TCGA dataset did not exhibit a significant difference in *XRCC4* expression. A previous detailed report suggests that LIG4 protein levels are upregulated in colon cancer tissues and mediate Wnt/beta-catenin signaling induced radioresistance (Jun et al., 2016). In another study, quantitative RT–PCR in 61 paired normal colon and 393 CRCs demonstrated *LIG4* downregulation in colon cancer tissues, which was further associated with its promoter hypermethylation (Kuhmann et al., 2014). While our results also suggest consistent downregulation of *LIG4* mRNA expression in tumor cells, proteomic analysis displayed higher *LIG4* levels in colon tumors. Furthermore, we observed higher expression of *LIG4* in advanced stage tumors and male patients. Association of *LIG4* mRNA expression with its protein levels and gender has not been reported and requires further exploration.

**FIGURE 9:**
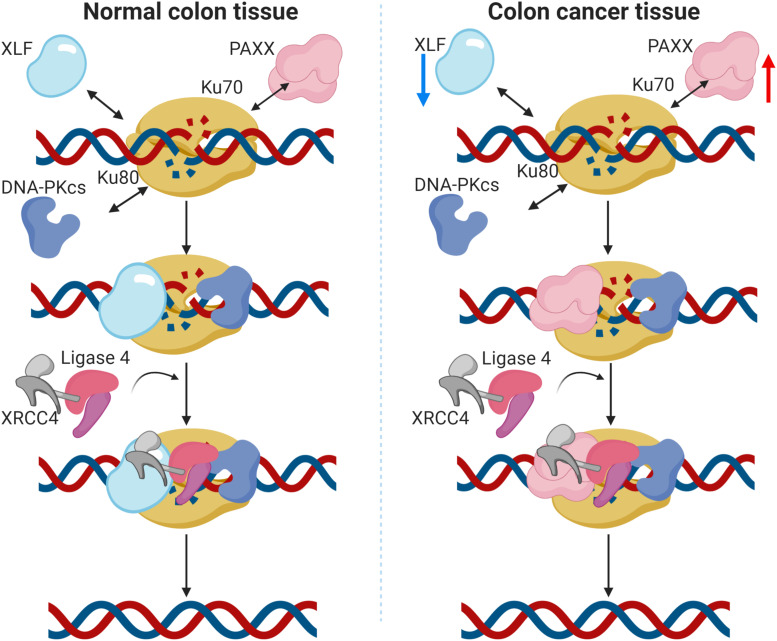
Graphical representation of the proposed function of PAXX in the NHEJ pathway in colon cancer. In normal colon tissues (**left** panel), DNA double strand breaks (DSBs) are actively identified by Ku70 and Ku80, followed by the recruitment of DNA-PKcs. The Ku/DNA-PKcs complex phosphorylates and recruits other accessory factors for DNA end processing. XRCC4 and XLF also bind to the DSB site and recruit DNA Ligase IV, which eventually seals the DSBs. PAXX has been demonstrated to work in the absence of XLF, as a backup in c-NHEJ repair (Tadi et al., 2016). In the case of colon cancer (**right** panel), protein levels of these proteins are altered. Notably, XLF is downregulated and PAXX is upregulated, suggesting PAXX may preferentially take over the XLF functions in colon cancer cells.

XLF (*NHEJ1*) was recently shown to enhance resistance to oxaliplatin and 5-fluorouracil in colorectal cancer cell lines (Liu et al., 2019). Association of higher XLF expression with drug resistance in hepatocellular carcinoma has also been reported (Yang and Wang, 2017). Contrary to these observations, we found consistent downregulation of XLF in colon cancer in our analysis, both at mRNA and the protein levels. Furthermore, its reduced mRNA expression was associated with the mucinous subtype, while no association was observed with the tumor stage. These results signify that although XLF is capable to induce drug resistance in CRC cells, its expression is nevertheless, downregulated in colon cancer. Intriguingly, we observed that *NHEJ1* expression is negatively correlated with *PAXX* expression, and PAXX was observed to be consistently overexpressed in colon tumors compared to the normal tissues, both at the mRNA and protein levels. Interestingly, PAXX and XLF are functionally redundant (Kumar et al., 2016; Tadi et al., 2016), and also exhibit synthetic lethality (Liu et al., 2017). These results suggest that *PAXX* may preferentially function over XLF in DSB repair in colon cancer, which has been graphically represented in Figure 9.

Our survival analysis revealed that among the NHEJ pathway genes analyzed in the current study, only *PAXX* emerged as an independent prognostic biomarker, while other *NHEJ1* genes did not display any prognostic significance. In concordance to *PAXX* overexpression observed in colon tumors, higher expression of *PAXX* was associated with poor OS and DSS. Further, the expression and prognostic value of *PAXX* did not display any association with the stage and MSI status. DNA methylation analysis revealed a negative correlation of *PAXX* expression with its promoter methylation and the extent of methylation in this gene was found to be lower in tumors compared to the normal colon. We conclude from these results that *PAXX* expression in colon cancer is at least partly under epigenetic control.

As our results suggest the utility of *PAXX* as a potential therapeutic target in colon cancer, we performed gene set enrichment analysis to further determine the association of *PAXX* expression with underlying oncogenic pathways in colon cancer. In agreement with its established role in DNA repair, *PAXX* associated genes were highly enriched in DNA repair and cell cycle related processes. Recently, Yang et al., reported that *PAXX* also plays an important role in the base excision repair pathway and *PAXX* deficient cells display higher sensitivity to temozolomide in glioma cells (Yang et al., 2018). These results collectively suggest that *PAXX* may play important roles in different DNA repair pathways as well and *PAXX* may serve as a novel therapeutic target for DNA repair in cancer cells. Much before the detailed functions of PAXX were determined, Meyer et al. reported the association of *PAXX* overexpression with rapid leukemia establishment in a mouse model of human acute lymphocytic leukemia xenograft, and shorter time to relapse in the corresponding patients (Meyer et al., 2011). Other pathways associated with higher *PAXX* expression, were related to cell metabolism, including higher oxidative phosphorylation and glycolytic pathway while UV response, *KRAS* signaling, and angiogenesis pathways were associated with lower *PAXX* expression. While pathway analysis in the present study revealed close associations of *PAXX* expression with several other oncogenic pathways as well, it requires further exploration to provide causal relationships between *PAXX* expression and alterations of these pathways. Nevertheless, we observed that several immune system associated pathways including inflammatory response, TGF beta signaling, and complement pathway were negatively associated with *PAXX* expression. Furthermore, *PAXX* expression exhibited a negative association with the abundance of immune cells in the colon tumor microenvironment, which suggests its association with reduced overall infiltration of immune cells in colon cancer. Interestingly, reduced tumor inflammatory infiltrate is generally associated with poor prognosis in colorectal cancers (Mei et al., 2014). Thus our study provides novel insights into NHEJ pathway status in colon cancer and suggests the potential utility of PAXX as a novel prognostic marker and a therapeutic target in colon cancer.

## Data Availability Statement

All datasets presented in this study are included in the article/Supplementary Material.

## Ethics Statement

Ethical review and approval was not required for the study on human participants in accordance with the local legislation and institutional requirements.

## Author Contributions

MA conceptualized the study. SC supervised the study and provided infrastructure to carry out this work. MA, SK, JS, and AC performed the data curation, interpretation, and statistical analysis. MA and SK wrote the original manuscript. AC and SC reviewed and edited the manuscript. All the authors approved the final manuscript.

## Conflict of Interest

The authors declare that the research was conducted in the absence of any commercial or financial relationships that could be construed as a potential conflict of interest.

## Footnotes

1 https://www.oncomine.org/resource/login.html

2 https://xena.ucsc.edu/

3 https://www.cbioportal.org/

4 http://ualcan.path.uab.edu/

5 https://mexpress.be/

6 https://www.gsea-msigdb.org/

7 https://bioplex.hms.harvard.edu

8 http://cis.hku.hk/software.html
