# Supplementary material for: PAXX, Not NHEJ1 Is an Independent Prognosticator in Colon Cancer
Source: Front Mol Biosci. 2020 Oct 23;7:584053. doi: 10.3389/fmolb.2020.584053 (PMC7649742; doi:10.3389/fmolb.2020.584053)
Supplement: Supplementary file 1 [file Table_1.docx]

Supplementary Material

**Supplementary Table 1:** Correlation matrix of NHEJ pathway genes based on Spearman’s correlations value in TCGA-COAD dataset.

|  | **XRCC6** | **XRCC5** | **PRKDC** | **XRCC4** | **LIG4** | **NHEJ1** | **PAXX** |
| --- | --- | --- | --- | --- | --- | --- | --- |
| **XRCC6** | 1 | 0.4469757 | 0.1975668 | 0.2405135 | -0.07580873 | 0.1320411 | 0.03493542 |
| **XRCC5** | 0.4469757 | 1 | 0.3344423 | 0.4320771 | 0.3029311 | 0.11986 | -0.1243471 |
| **PRKDC** | 0.1975668 | 0.3344423 | 1 | 0.24378 | 0.3130476 | 0.265947 | -0.2228298 |
| **XRCC4** | 0.2405135 | 0.4320771 | 0.24378 | 1 | 0.50295 | 0.0488774 | -0.1517053 |
| **LIG4** | -0.07580873 | 0.3029311 | 0.3130476 | 0.50295 | 1 | 0.05319917 | -0.2898128 |
| **NHEJ1** | 0.1320411 | 0.11986 | 0.265947 | 0.0488774 | 0.05319917 | 1 | -0.1648714 |
| **PAXX** | 0.03493542 | -0.1243471 | -0.2228298 | -0.1517053 | -0.2898128 | -0.1648714 | 1 |


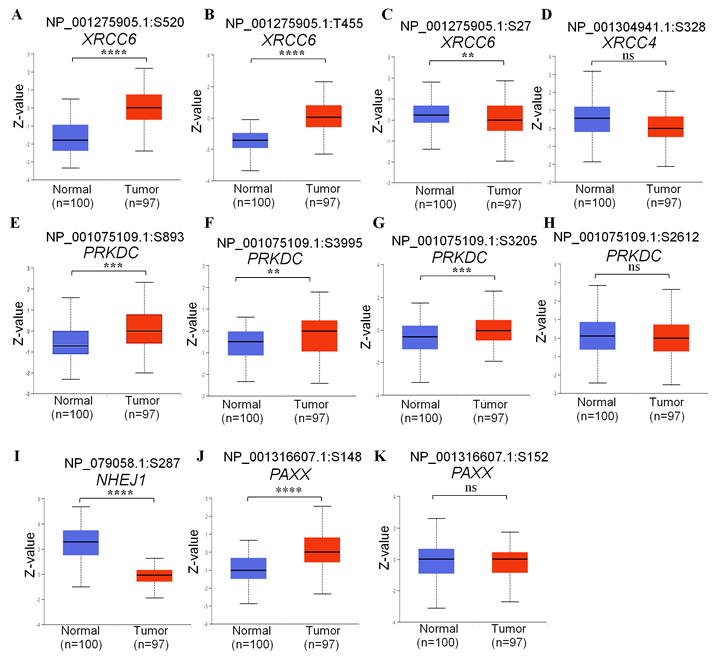
**Supplementary figure S1**: Comparison of phosphorylated protein levels of NHEJ pathway genes in normal and colon cancer tissues from CPTAC study, including (A) XRCC6 Ser520, (B) XRCC6 Thr455, (C) XRCC6 Ser27, (D) XRCC4 Ser328, (E) PRKDC Ser893, (F) PRKDC Ser3995, (G) PRKDC Ser3205, (H) PRKDC Ser2612, (I) XLF Ser287, (J) PAXX Ser148, and (K) PAXX Ser152. The peptides referenced for phosphoprotein detection have been depicted on top of each figure panel. *p<0.05, **p<0.01,***p<0.001,****p<0.0001.


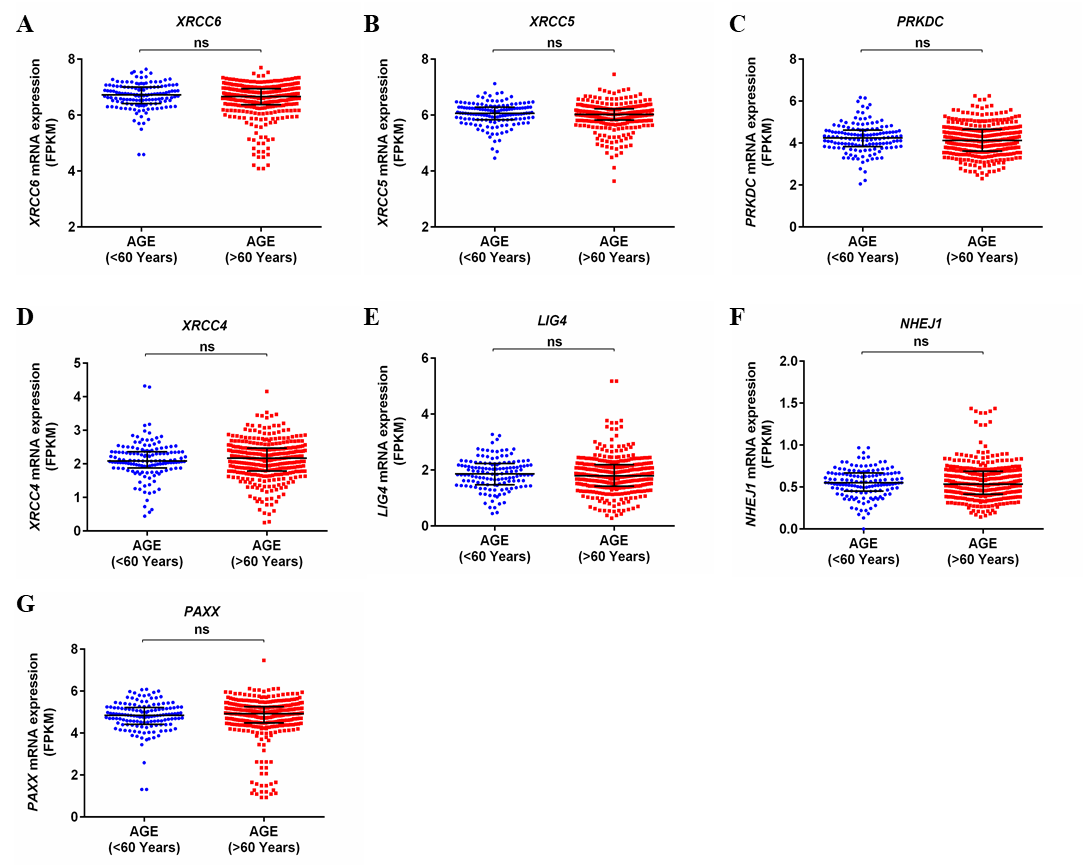


**Supplementary Figure S2:** Association of mRNA expression of NHEJ pathway genes from TCGA-COAD dataset with patient’s age. *p<0.05, **p<0.01,***p<0.001,****p<0.0001.

**
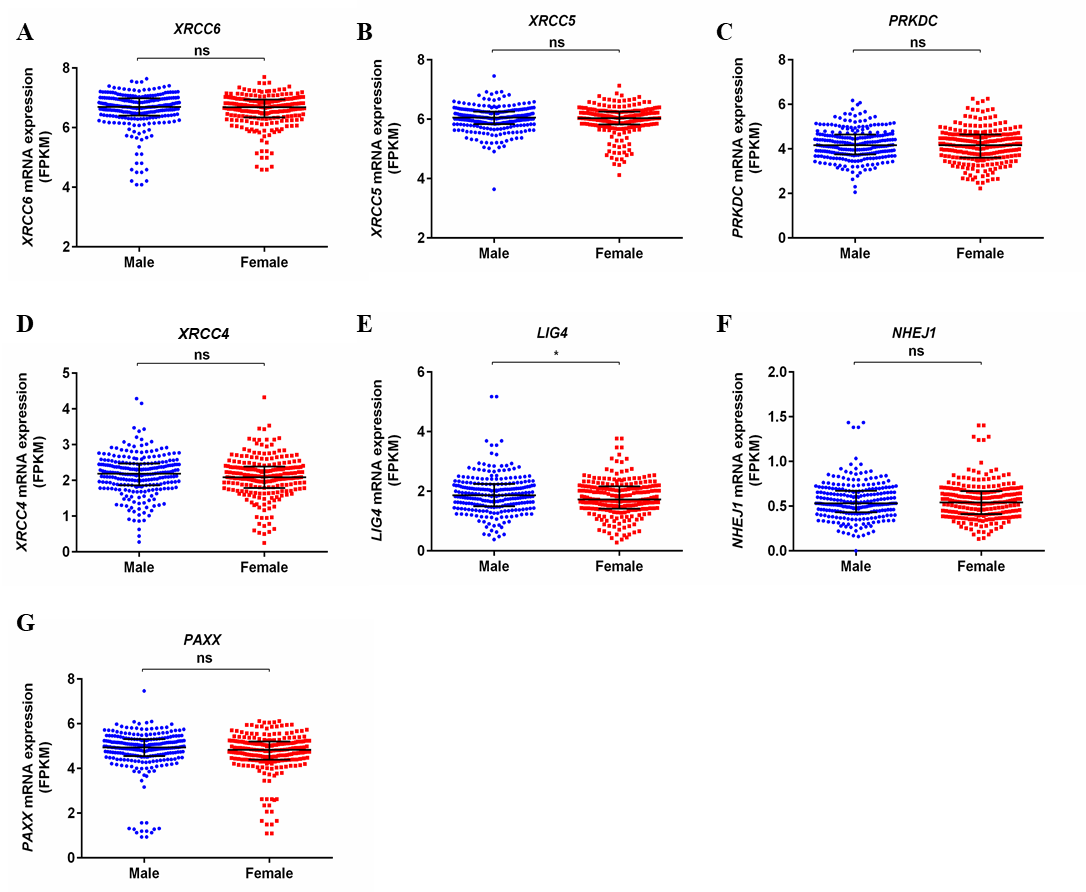
Supplementary Figure S3:** Association of mRNA expression of NHEJ pathway genes from TCGA-COAD dataset with gender. *p<0.05, **p<0.01,***p<0.001,****p<0.0001.


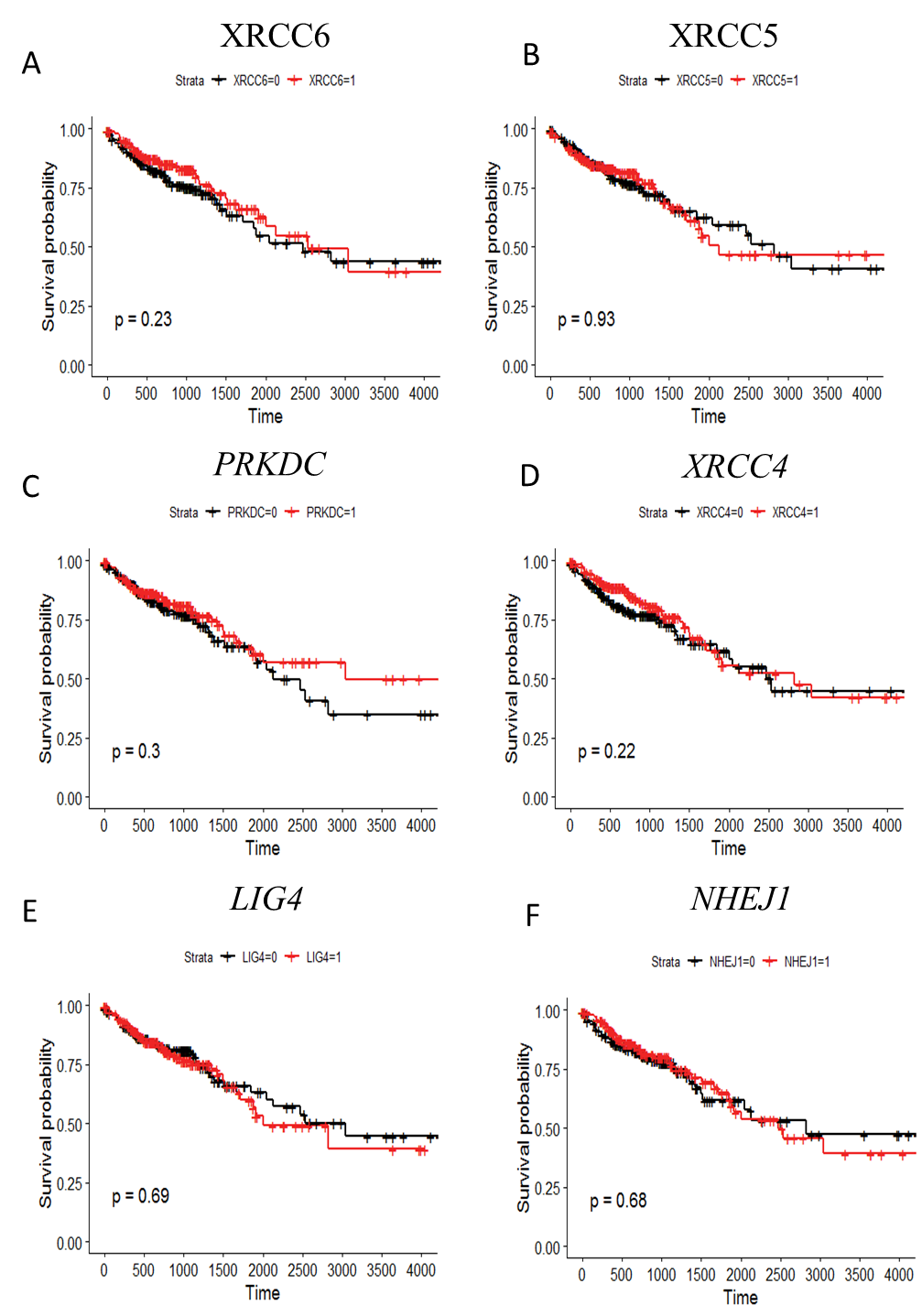


**Supplementary Figure S4:** Kaplan Meier survival curve for prognostic significance of NHEJ pathways genes for overall survival in colon cancer. (A) *XRCC6*, (B) *XRCC5*, (C) *PRKDC*, (D) *XRCC4*, (E) *LIG4*, and (F) *NHEJ1*. Patients were divided into two groups based on median expression value of respective genes from TCGA-COAD dataset. Survival probabilities are presented on the y-axis and time in days on the x-axis in all graphs. Log rank test p-values have been depicted in respective graphs.


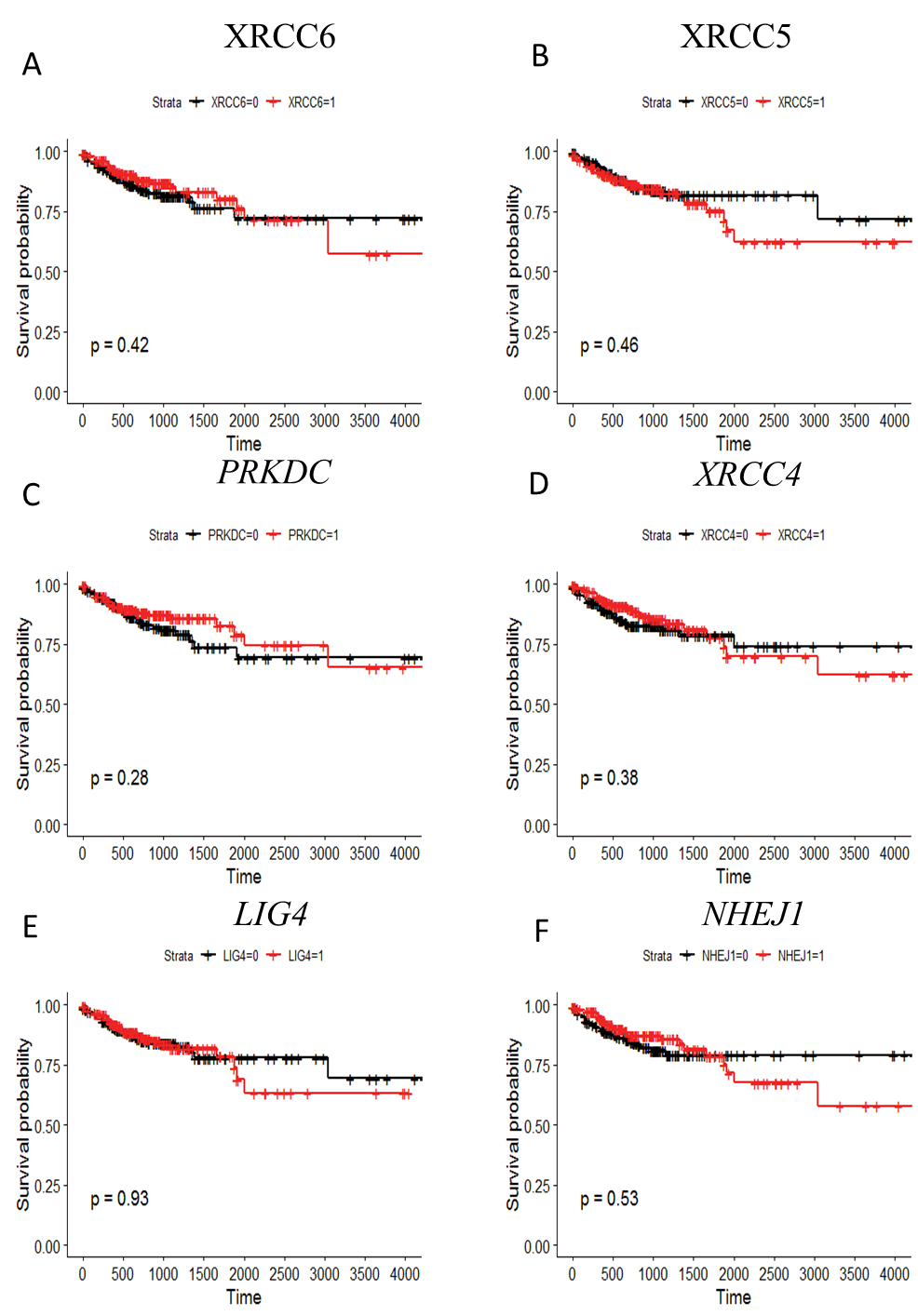


**Supplementary Figure S5:** Kaplan Meier survival curve for prognostic significance of NHEJ pathways genes for disease specific survival in colon cancer. (A) *XRCC6*, (B) *XRCC5*, (C) *PRKDC*, (D) *XRCC4*, (E) *LIG4*, and (F) *NHEJ1*. Patients were divided into two groups based on median expression value of respective genes from TCGA-COAD dataset. Survival probabilities are presented on the y-axis and time in days on the x-axis in all graphs. Log rank test p-values have been depicted in respective graphs.


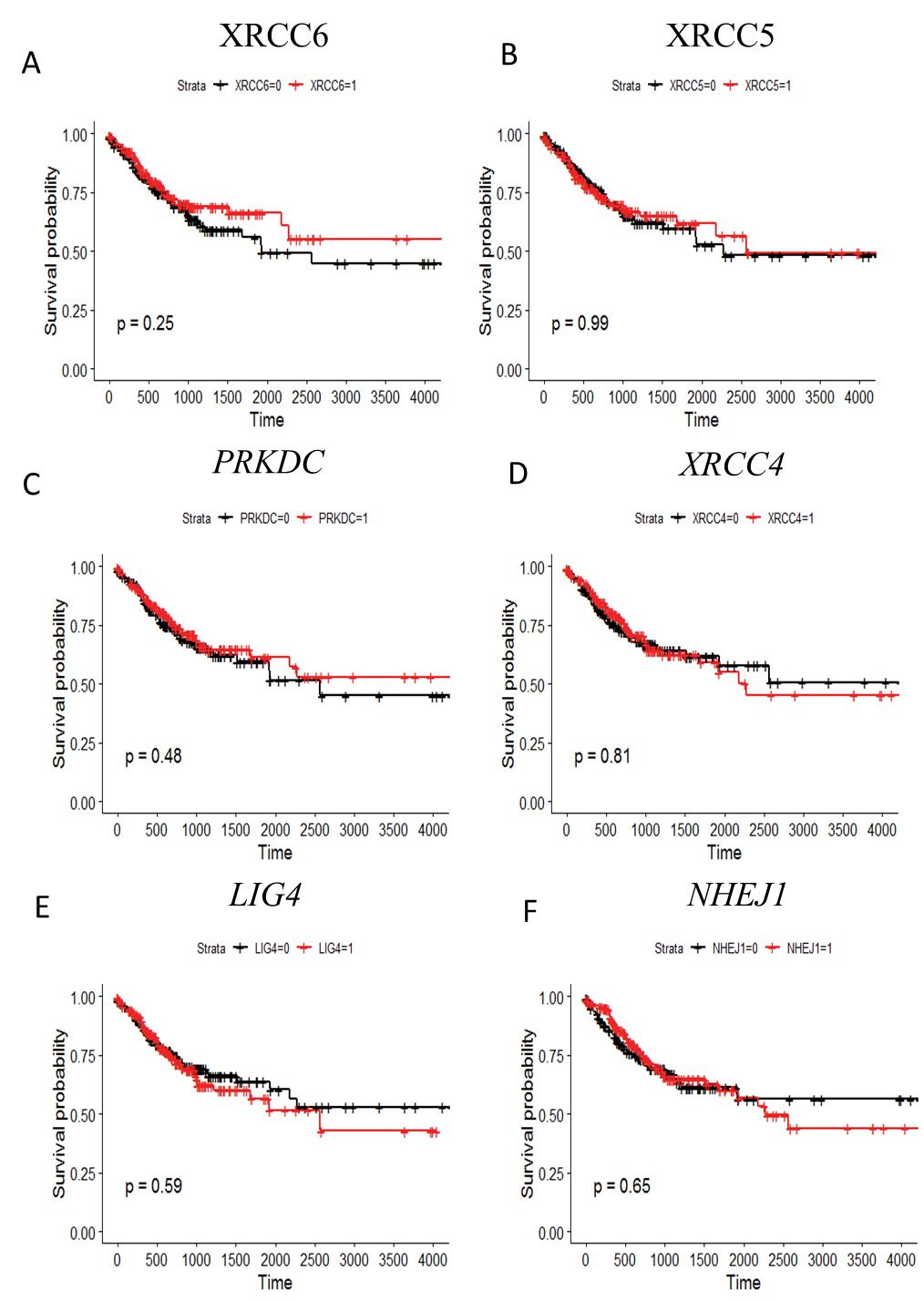


**Supplementary Figure S6:** Kaplan Meier survival curve for prognostic significance of NHEJ pathways genes for progression free interval in colon cancer. (A) *XRCC6*, (B) *XRCC5*, (C) *PRKDC*, (D) *XRCC4*, (E) *LIG4*, and (F) *NHEJ1*. Patients were divided into two groups based on median expression value of respective genes from TCGA-COAD dataset. Survival probabilities are presented on the y-axis and time in days on the x-axis in all graphs. Log rank test p-values have been depicted in respective graphs.


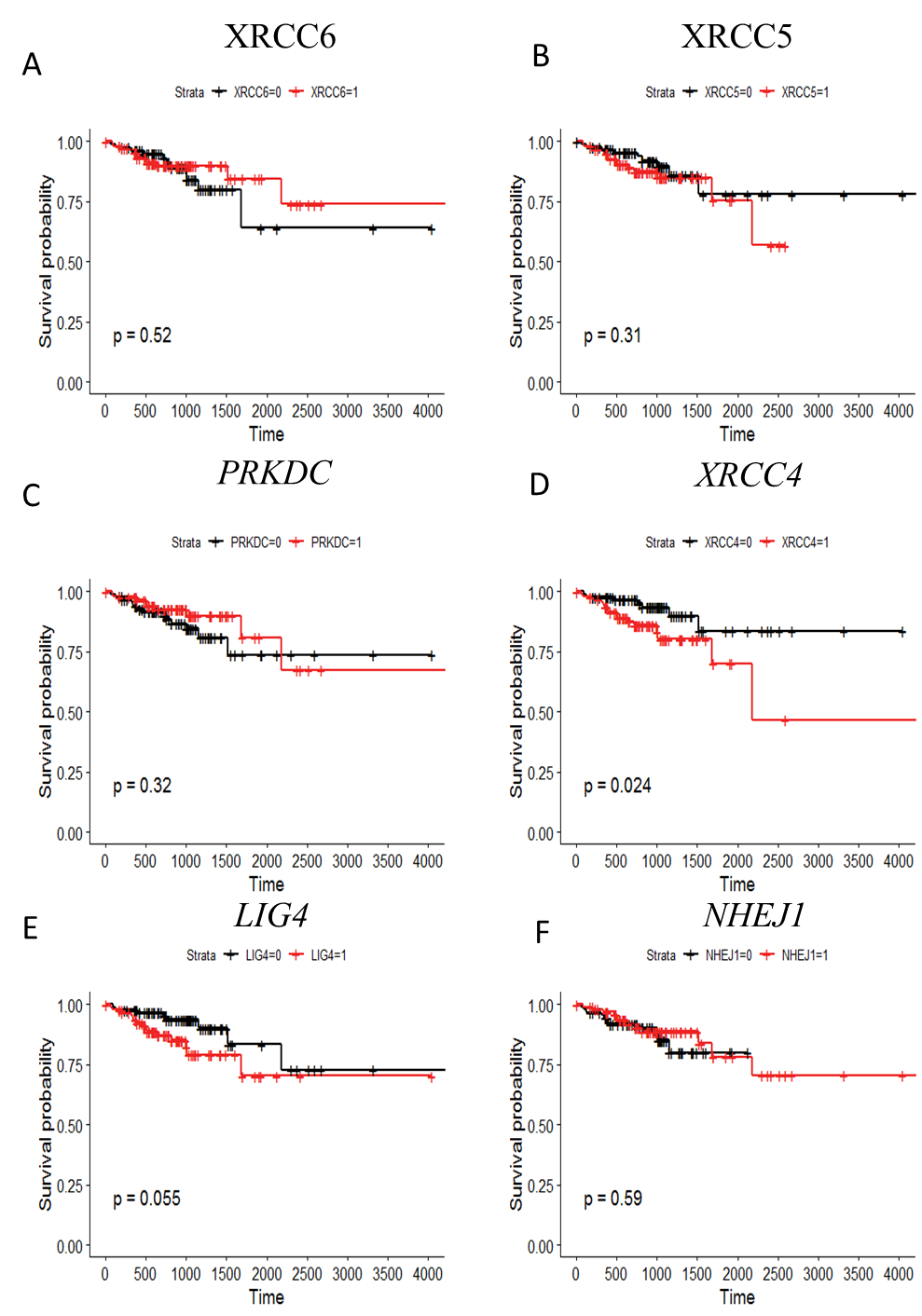


**Supplementary Figure S7:** Kaplan Meier survival curve for prognostic significance of NHEJ pathways genes for disease free interval in colon cancer. (A) *XRCC6*, (B) *XRCC5*, (C) *PRKDC*, (D) *XRCC4*, (E) *LIG4*, and (F) *NHEJ1*. Patients were divided into two groups based on median expression value of respective genes from TCGA-COAD dataset. Survival probabilities are presented on the y-axis and time in days on the x-axis in all graphs. Log rank test p-values have been depicted in respective graphs.


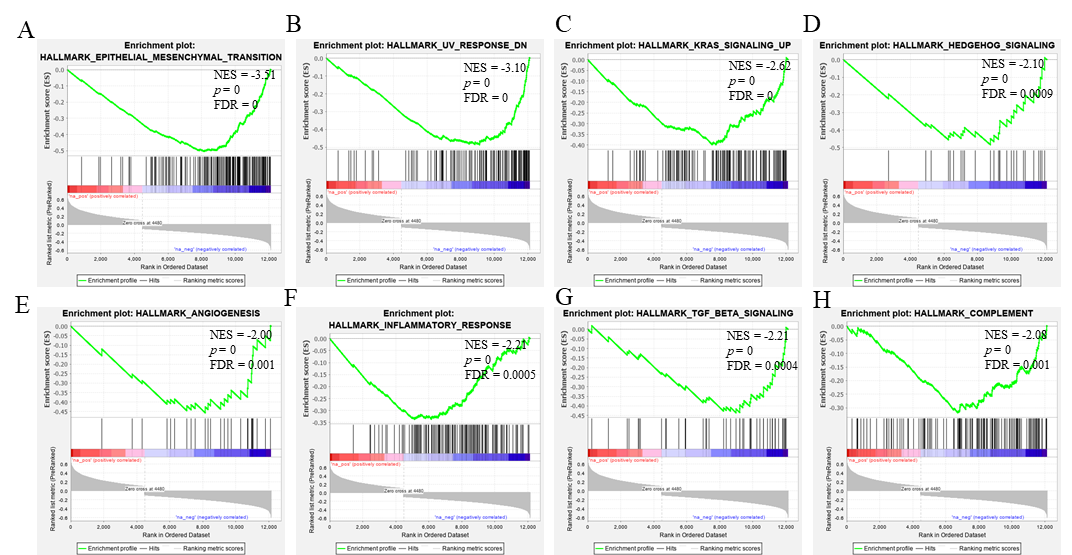


**Supplementary Figure S8:** Gene set enrichment analysis of *PAXX* correlated genes in the TCGA-COAD dataset. Each plot (A-H) depicts negatively enriched pathways of *PAXX* correlated genes with Normalized ES (NES), false discovery rate (FDR), and p-value depicted inside the respective pathway panel.


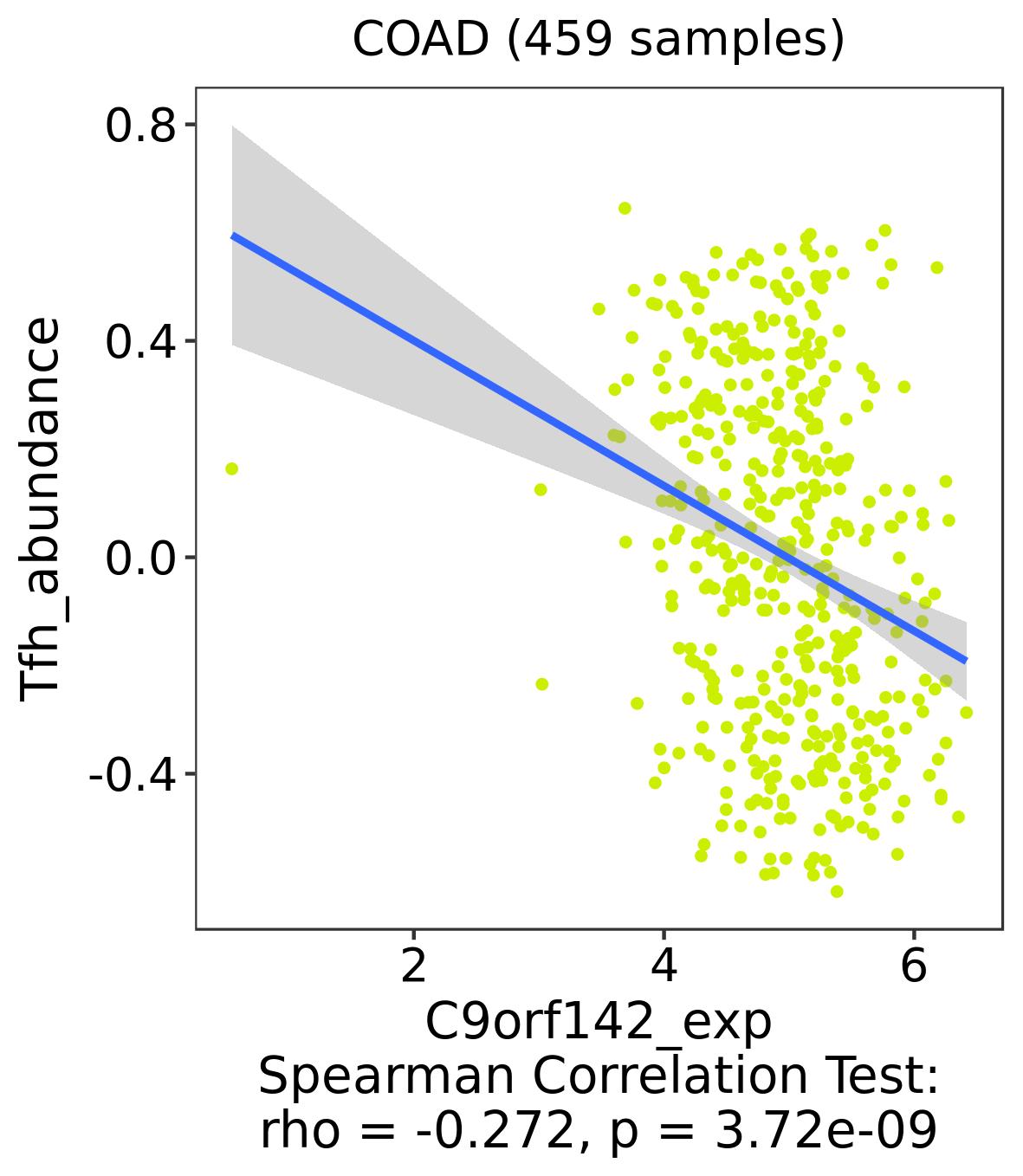

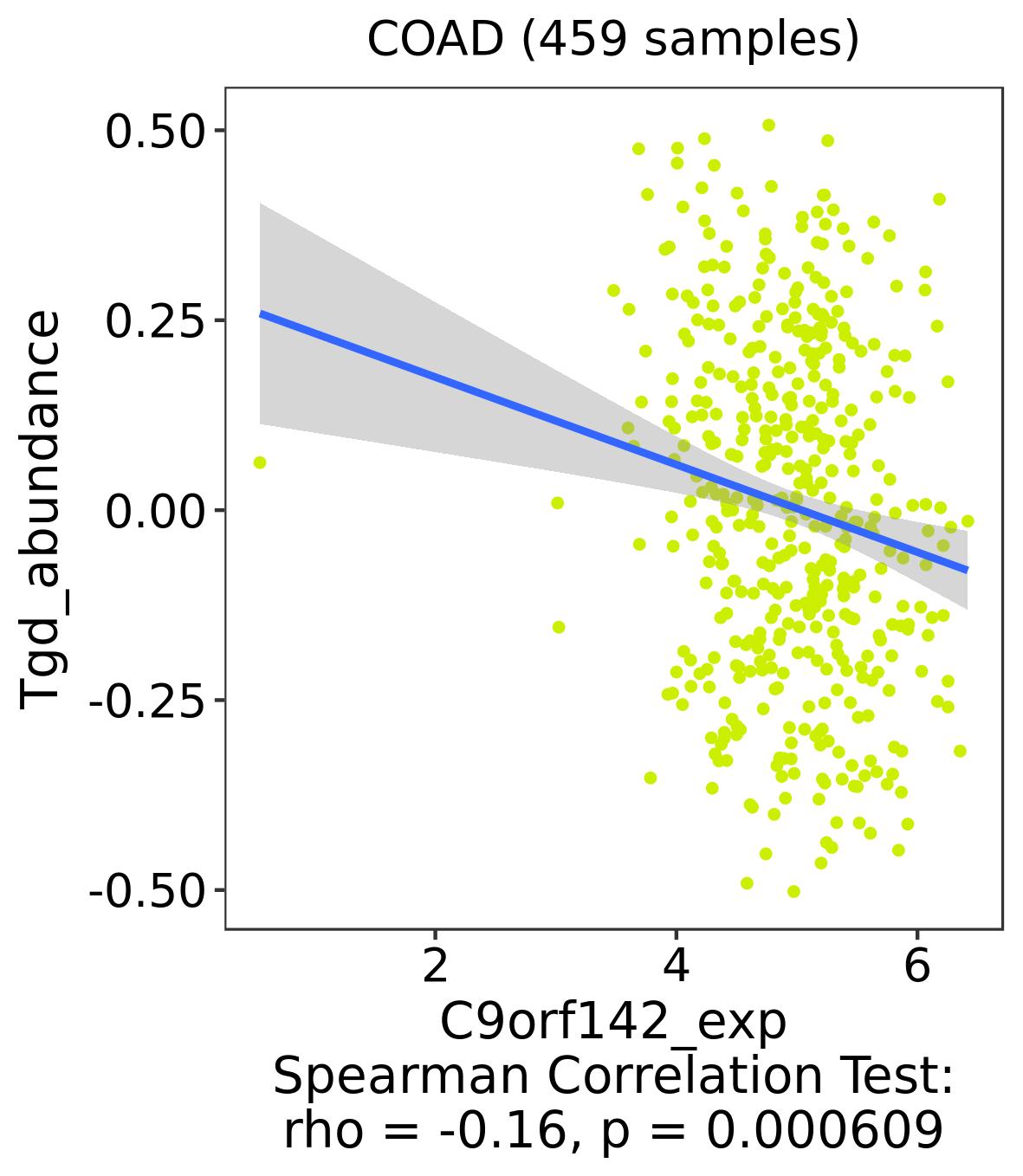

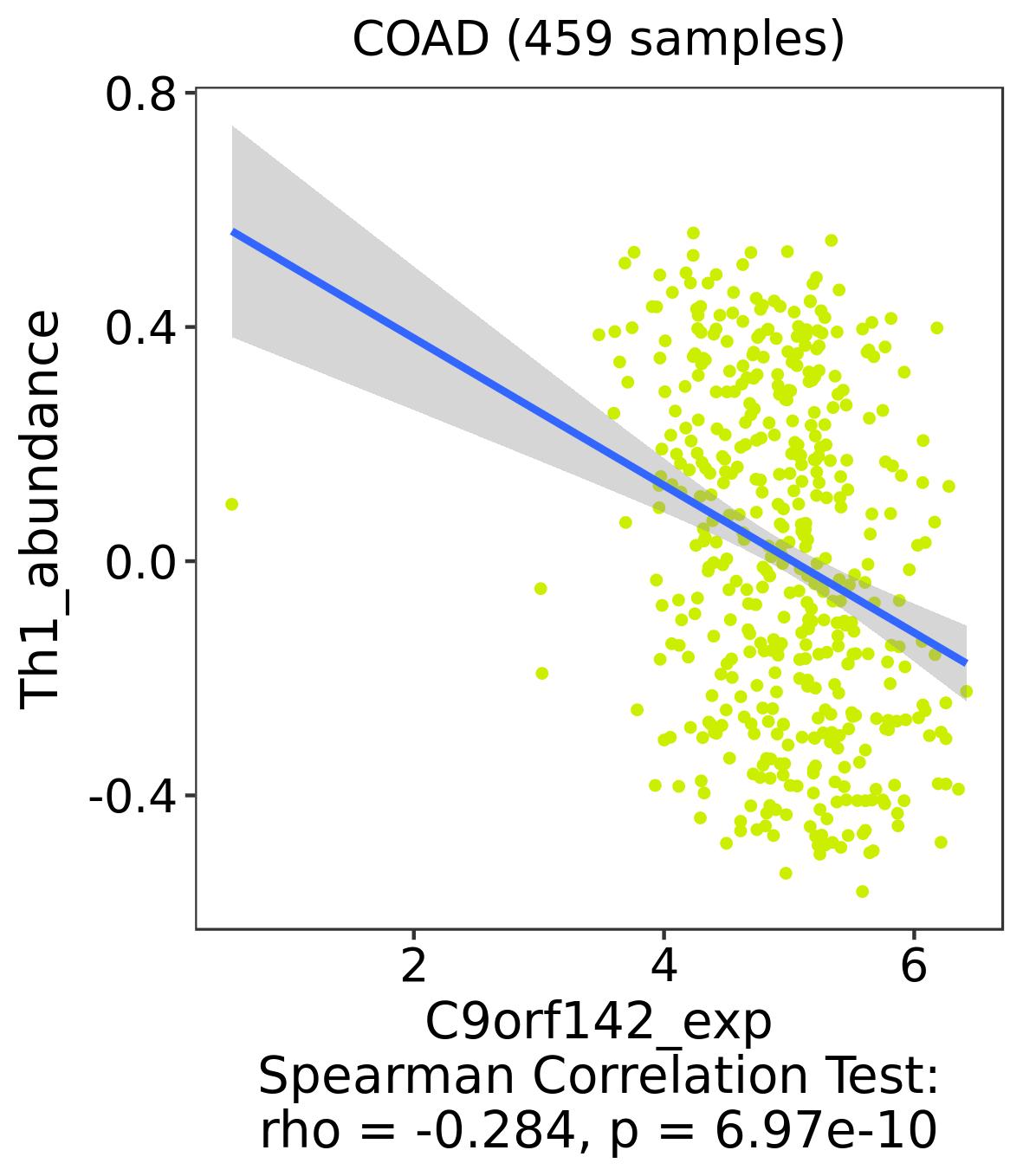

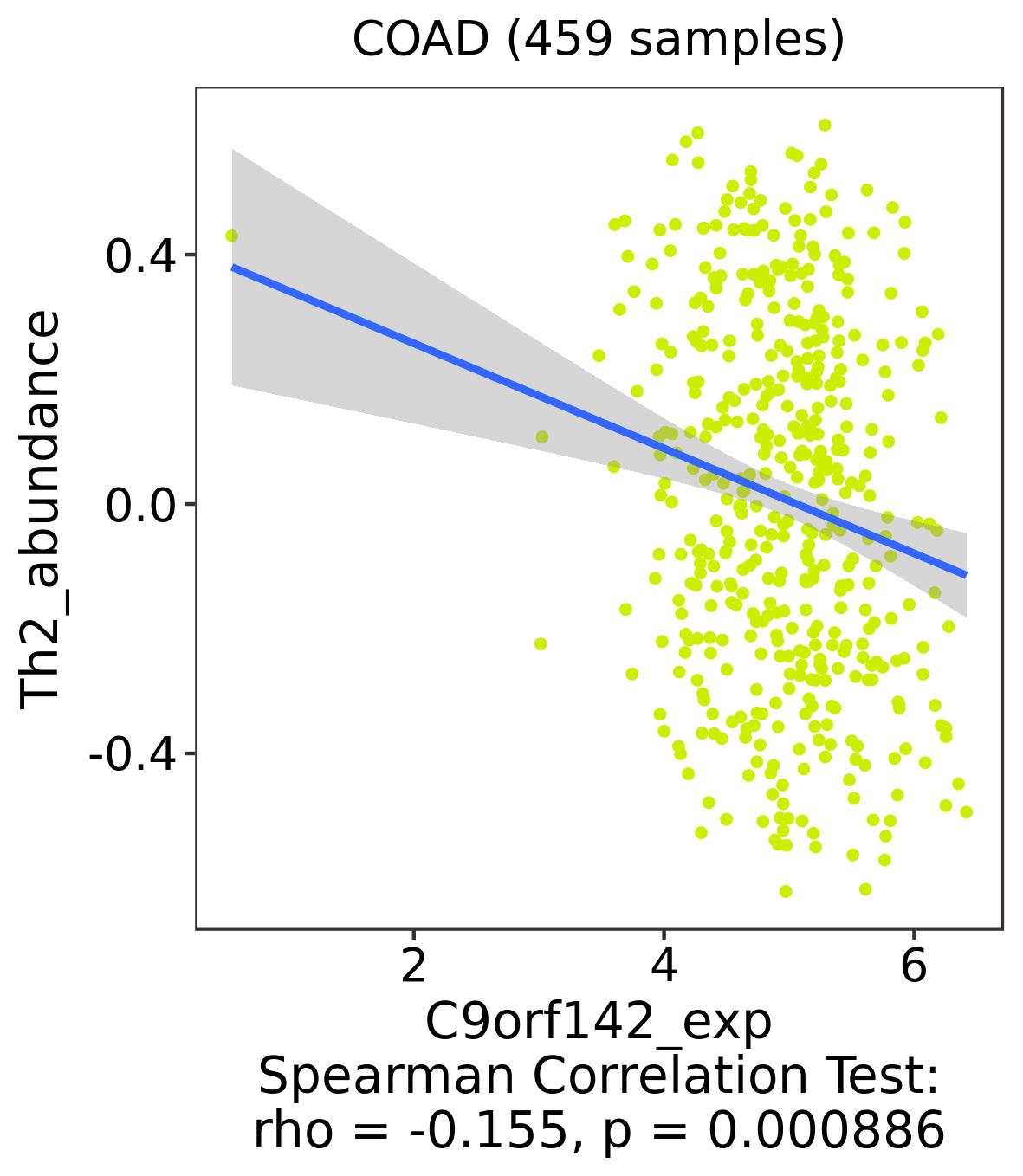

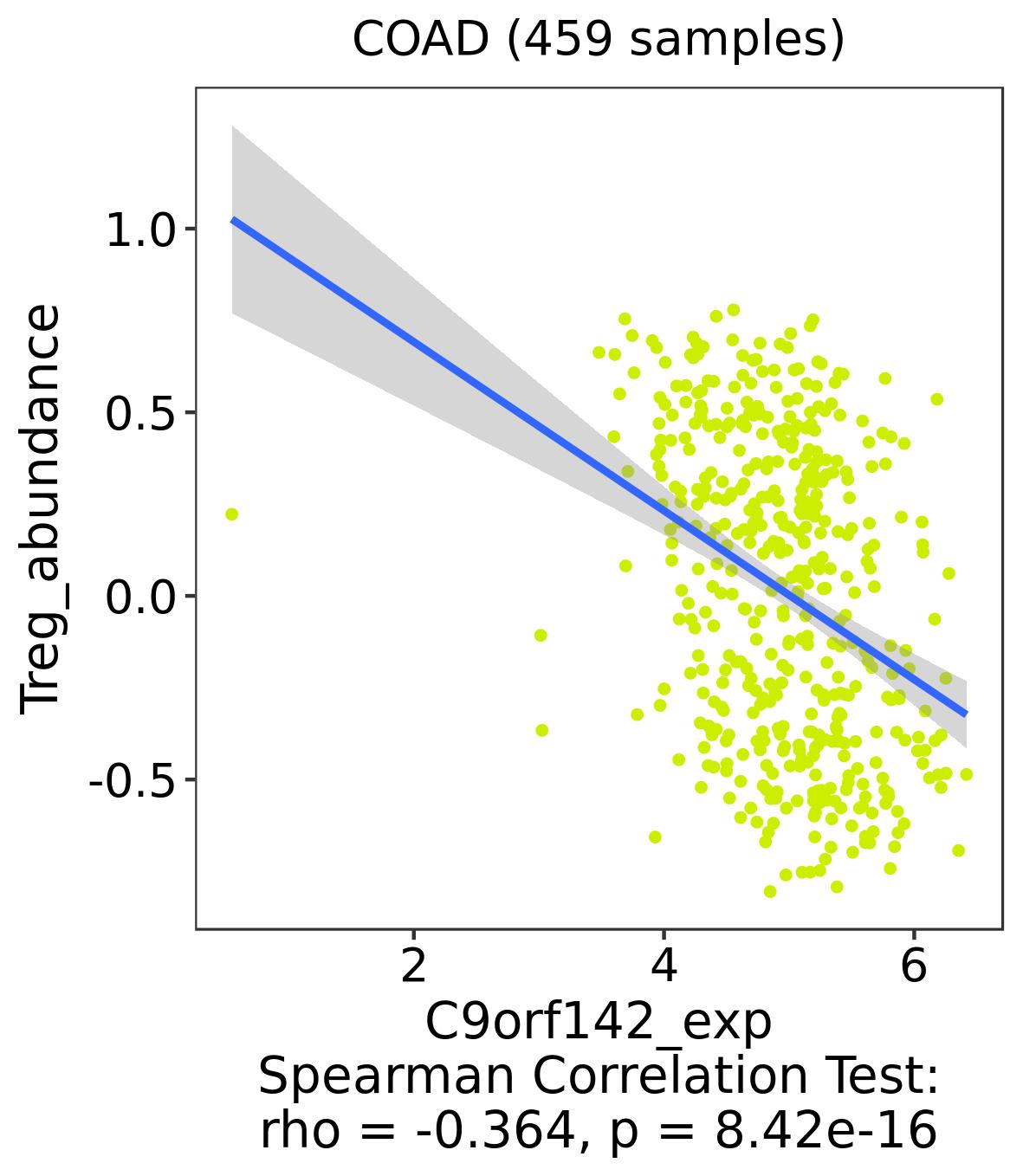

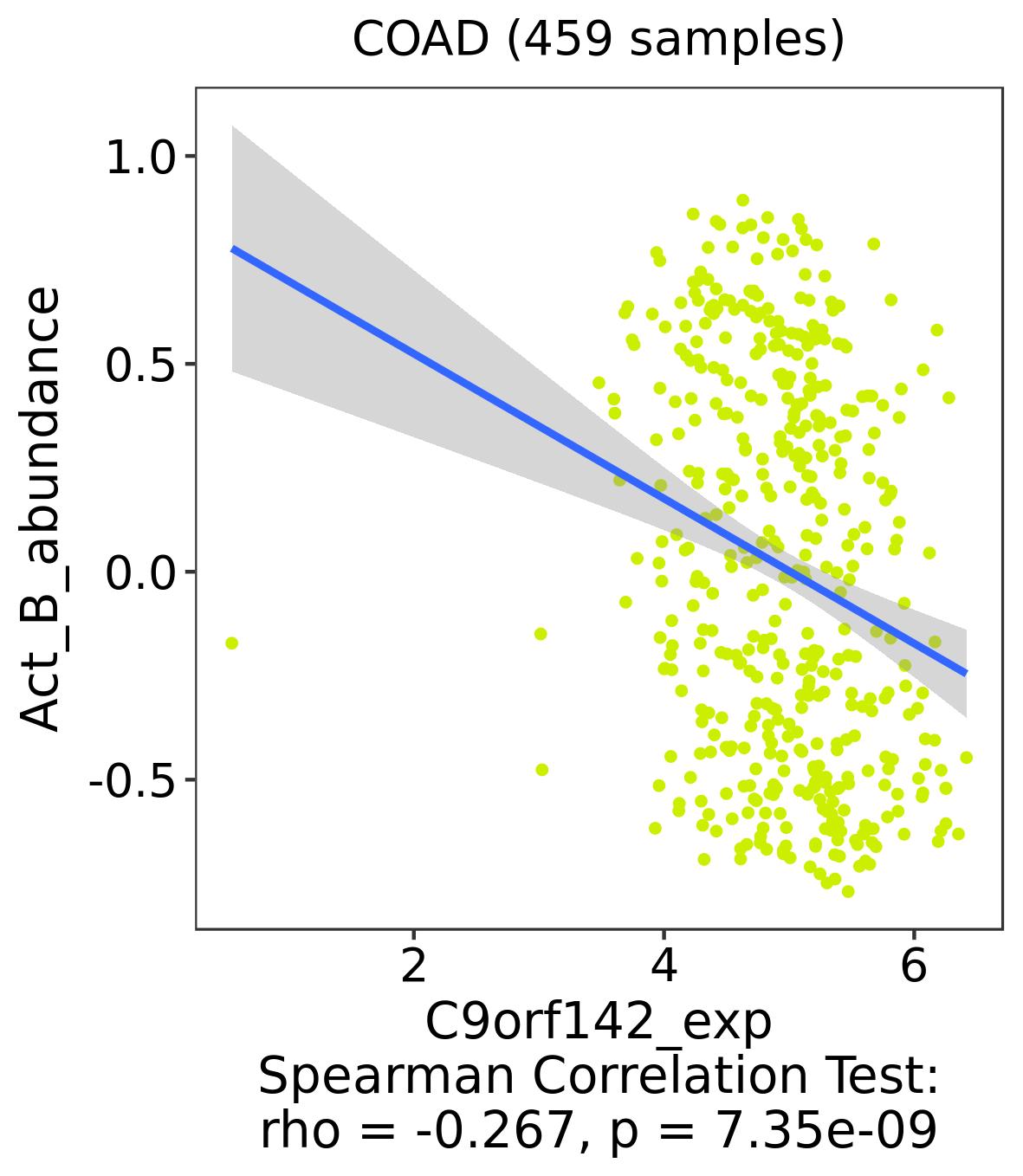

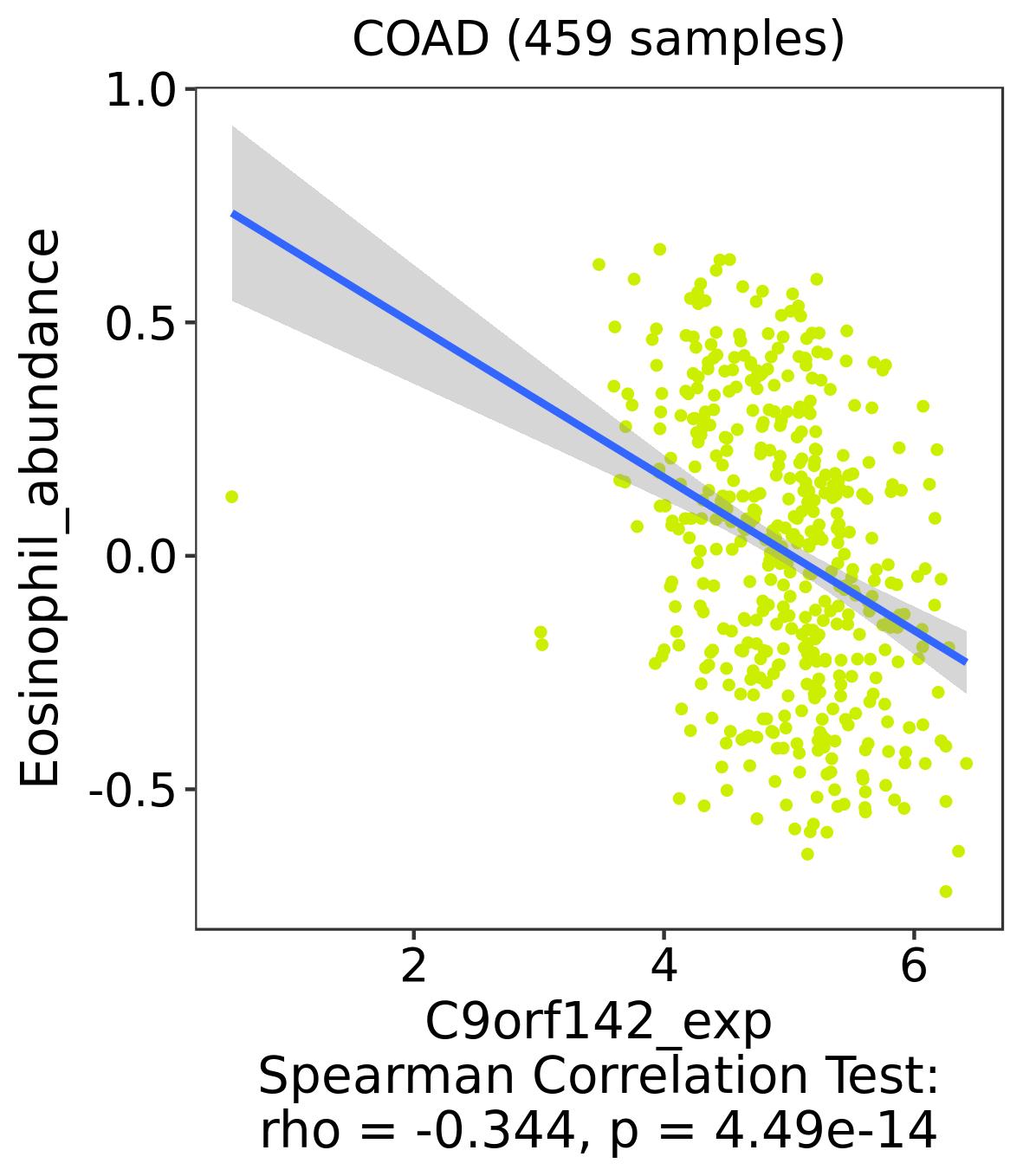

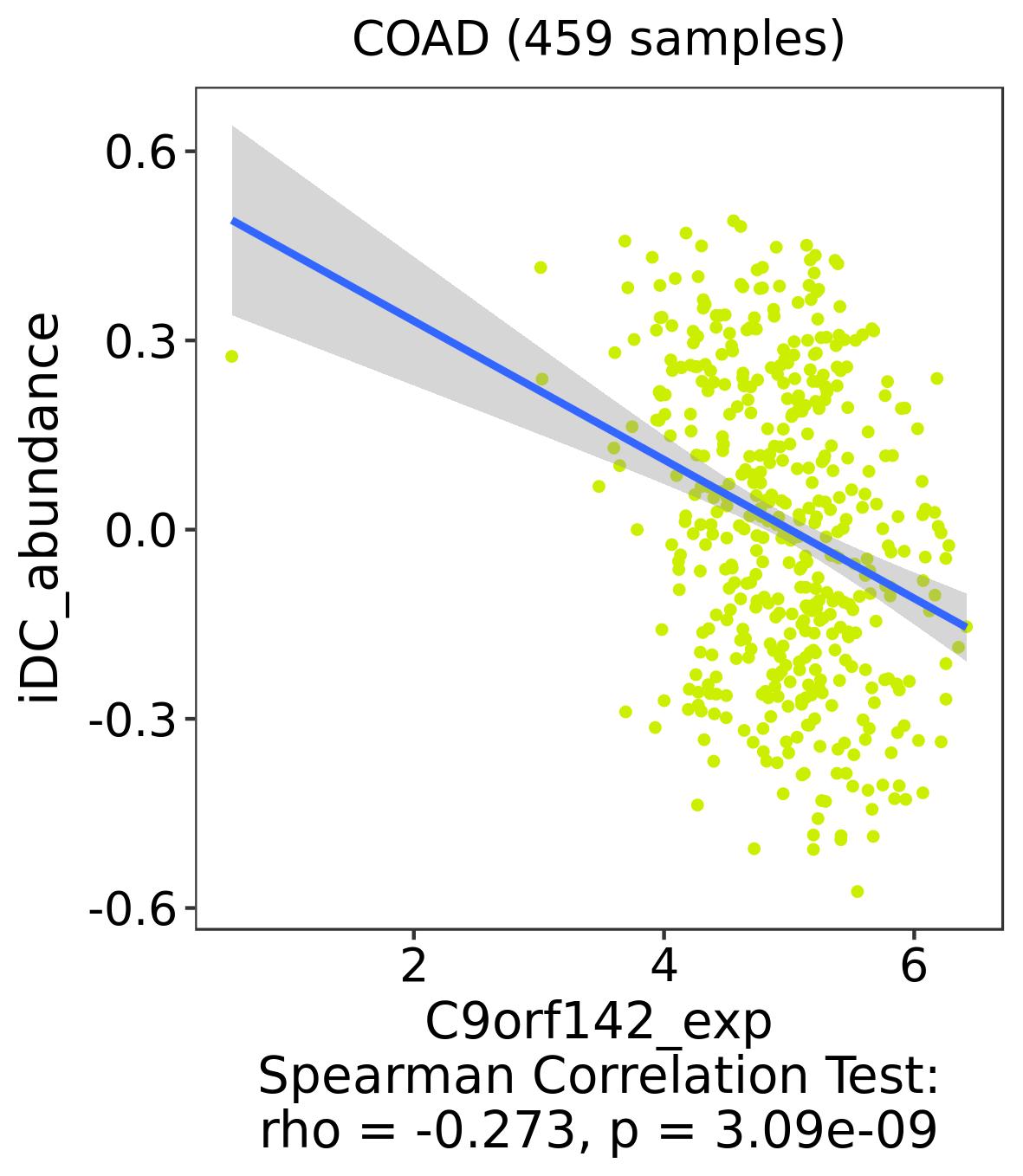

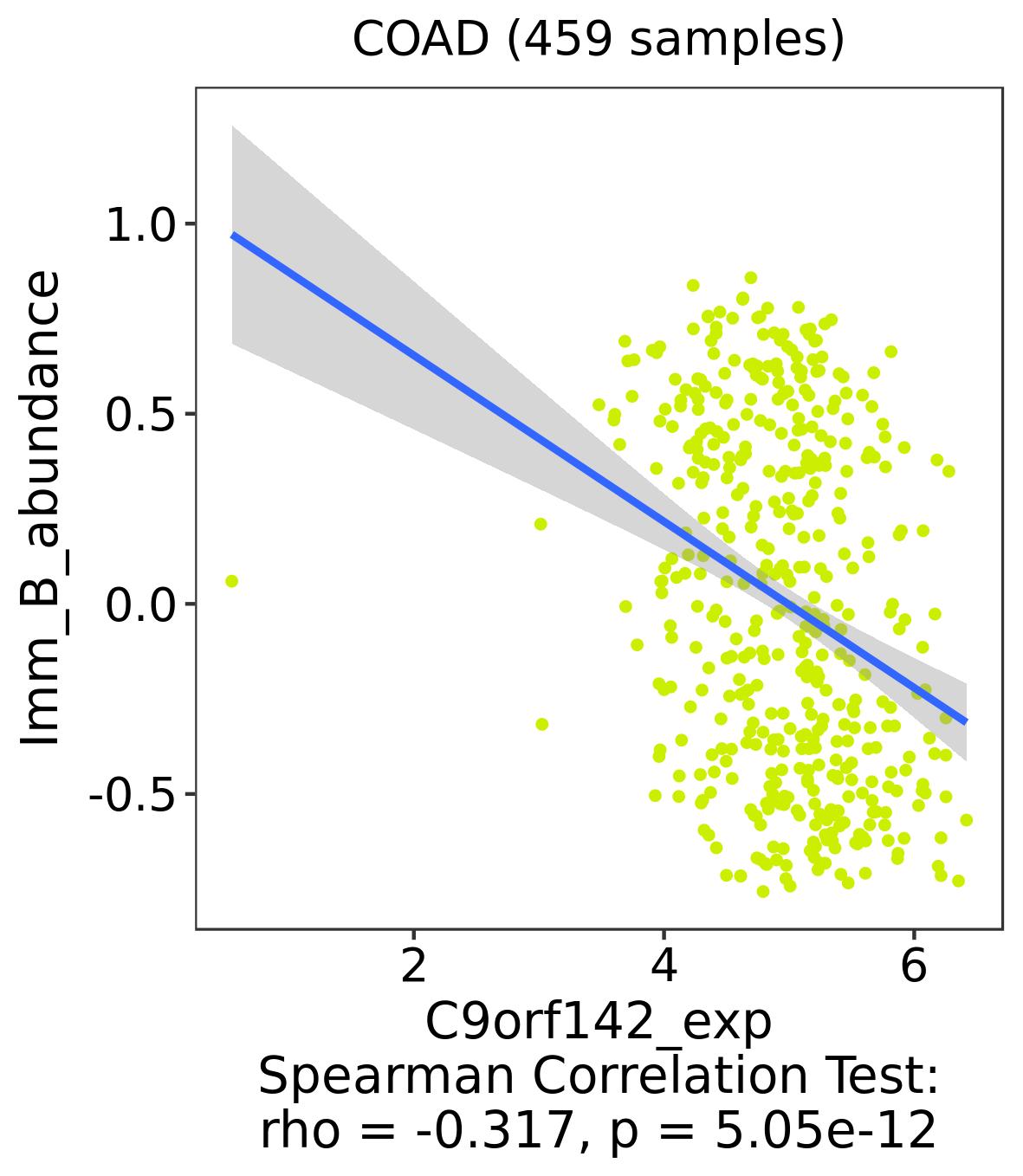

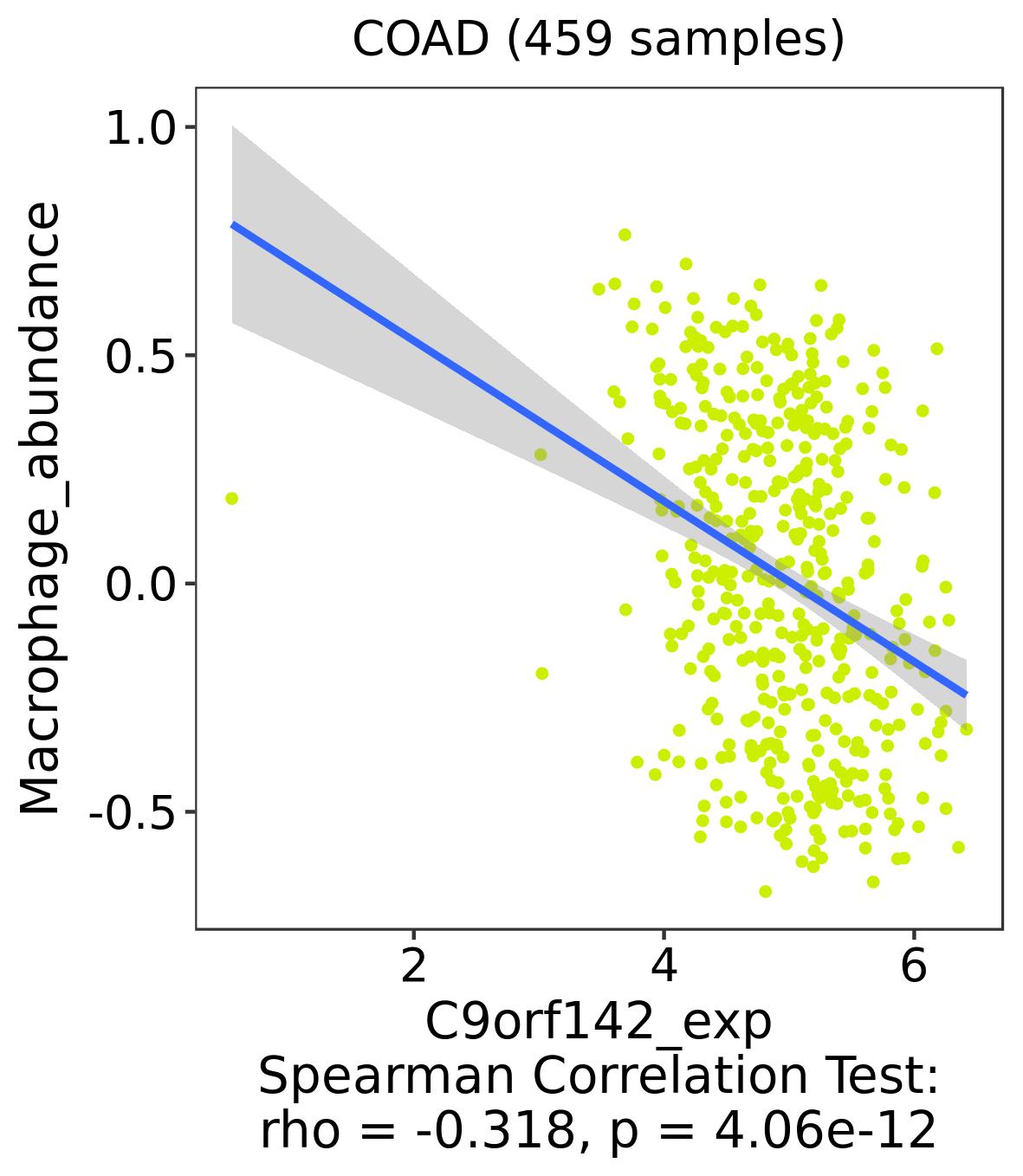

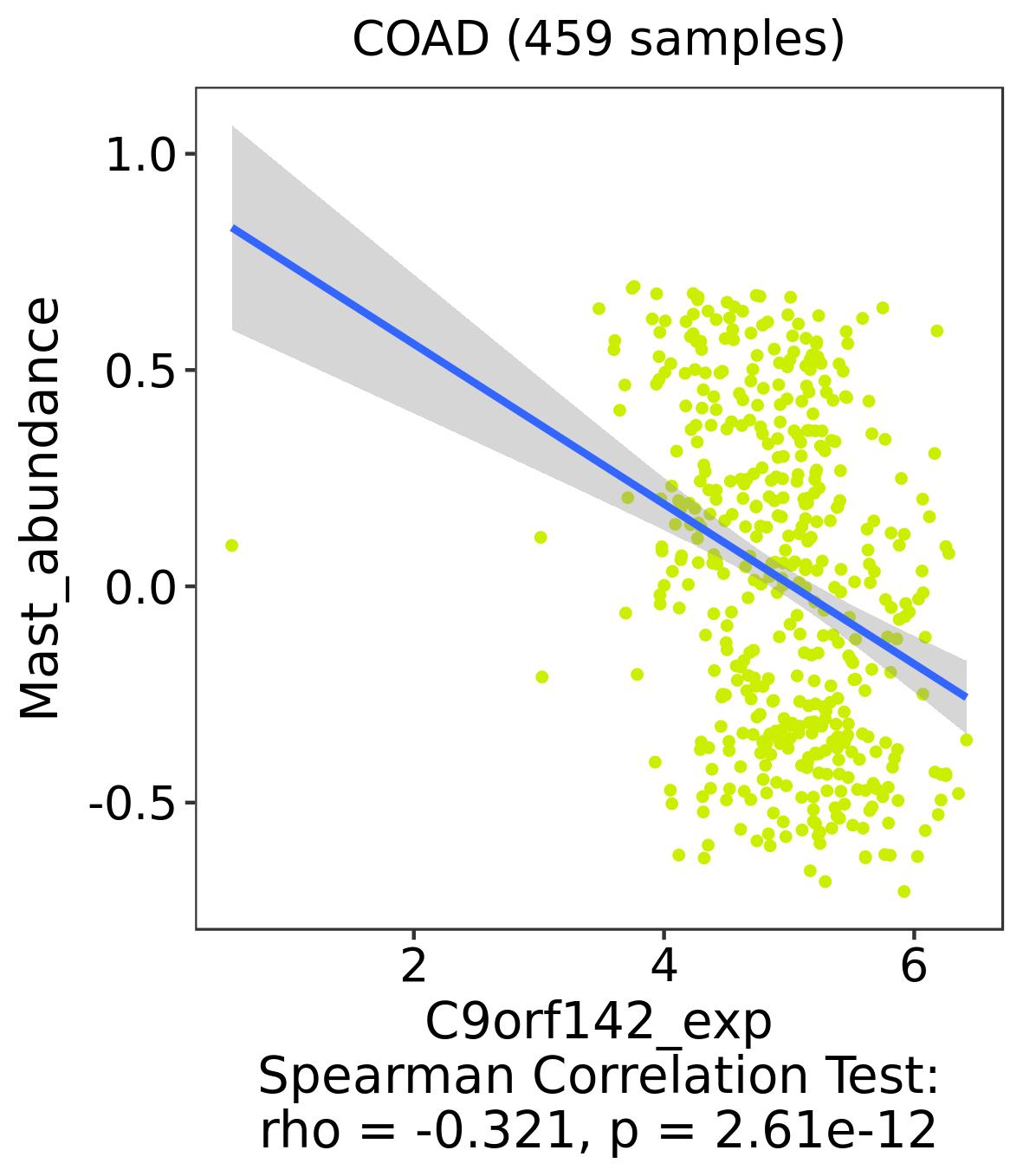

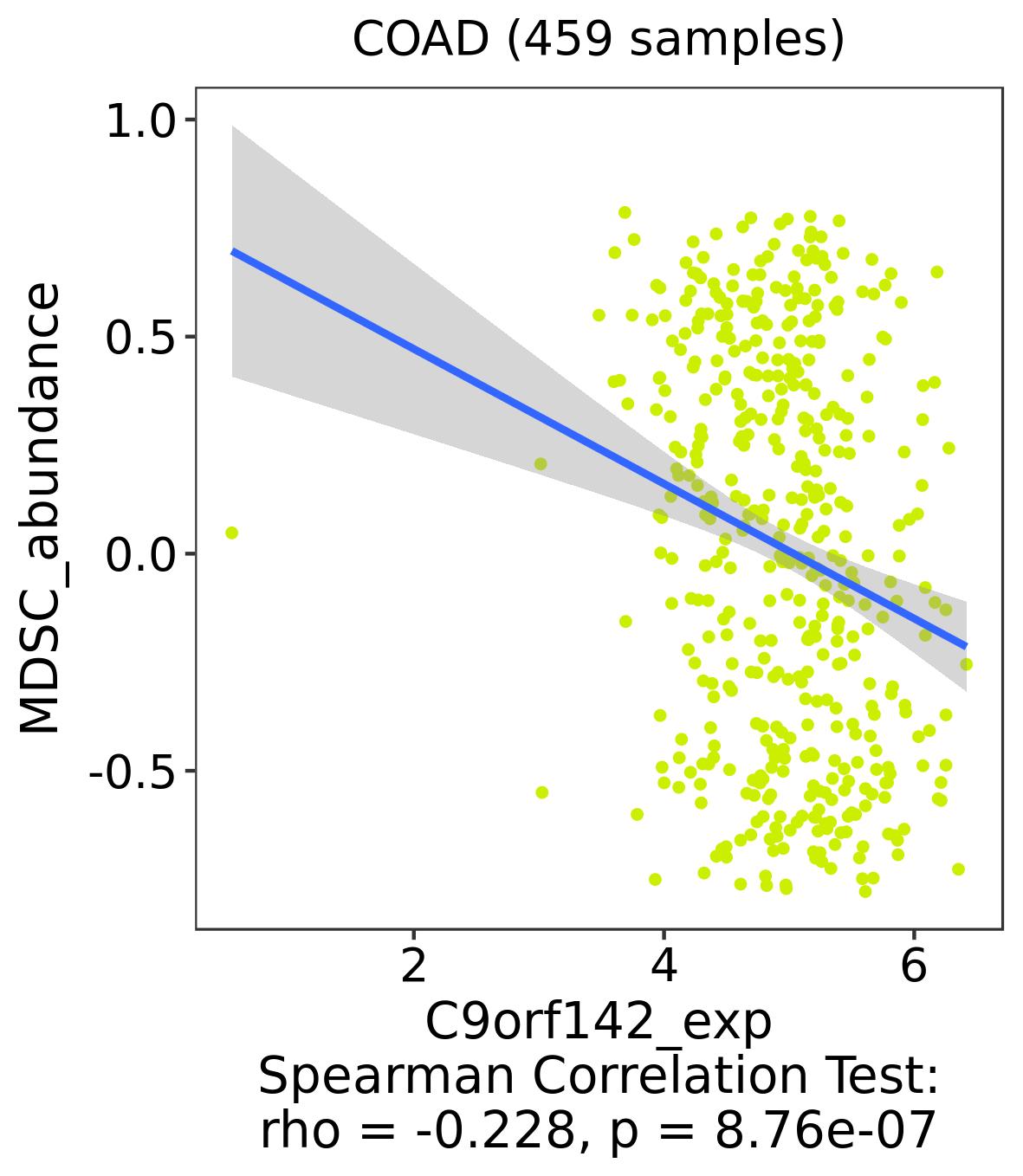

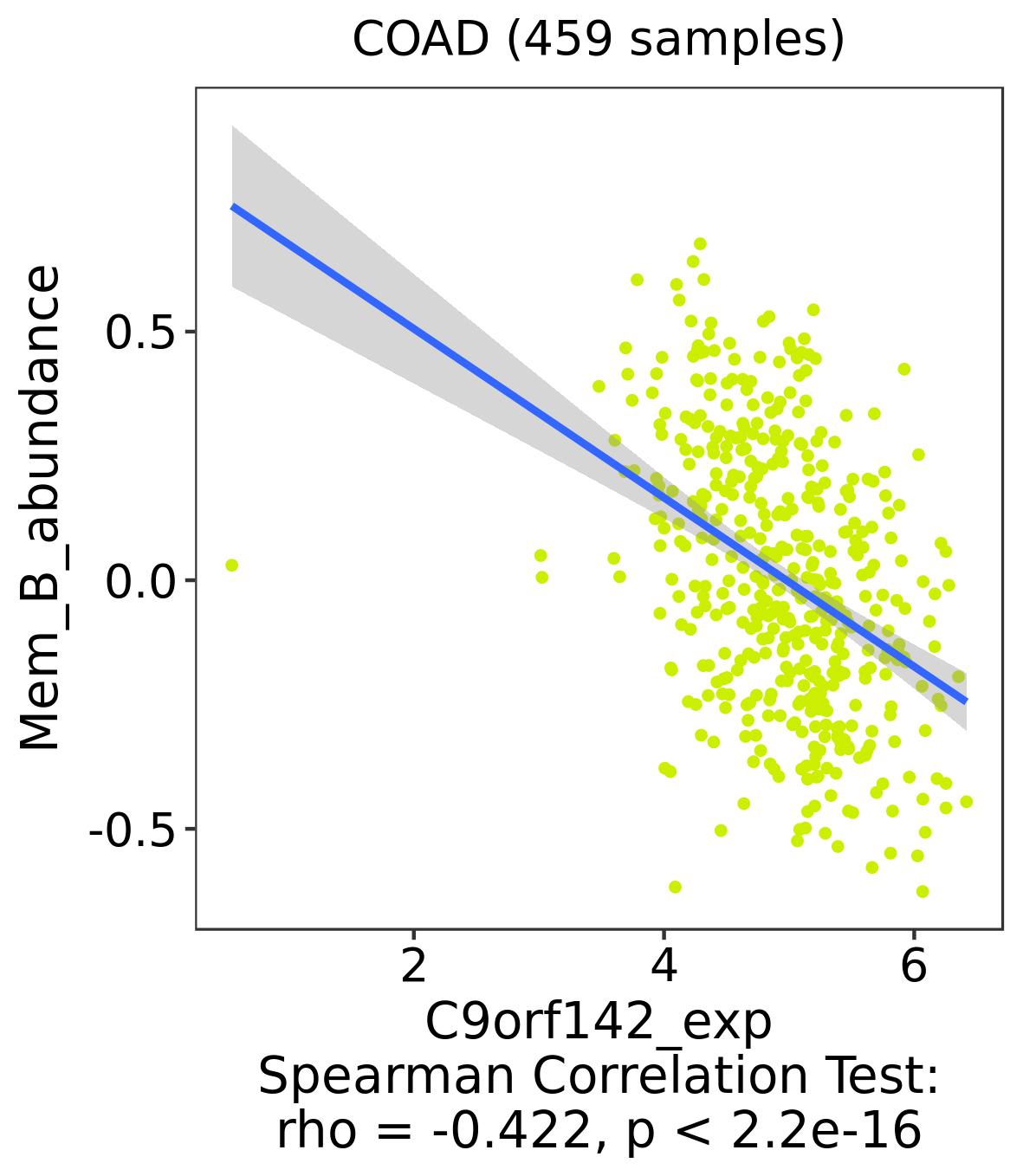

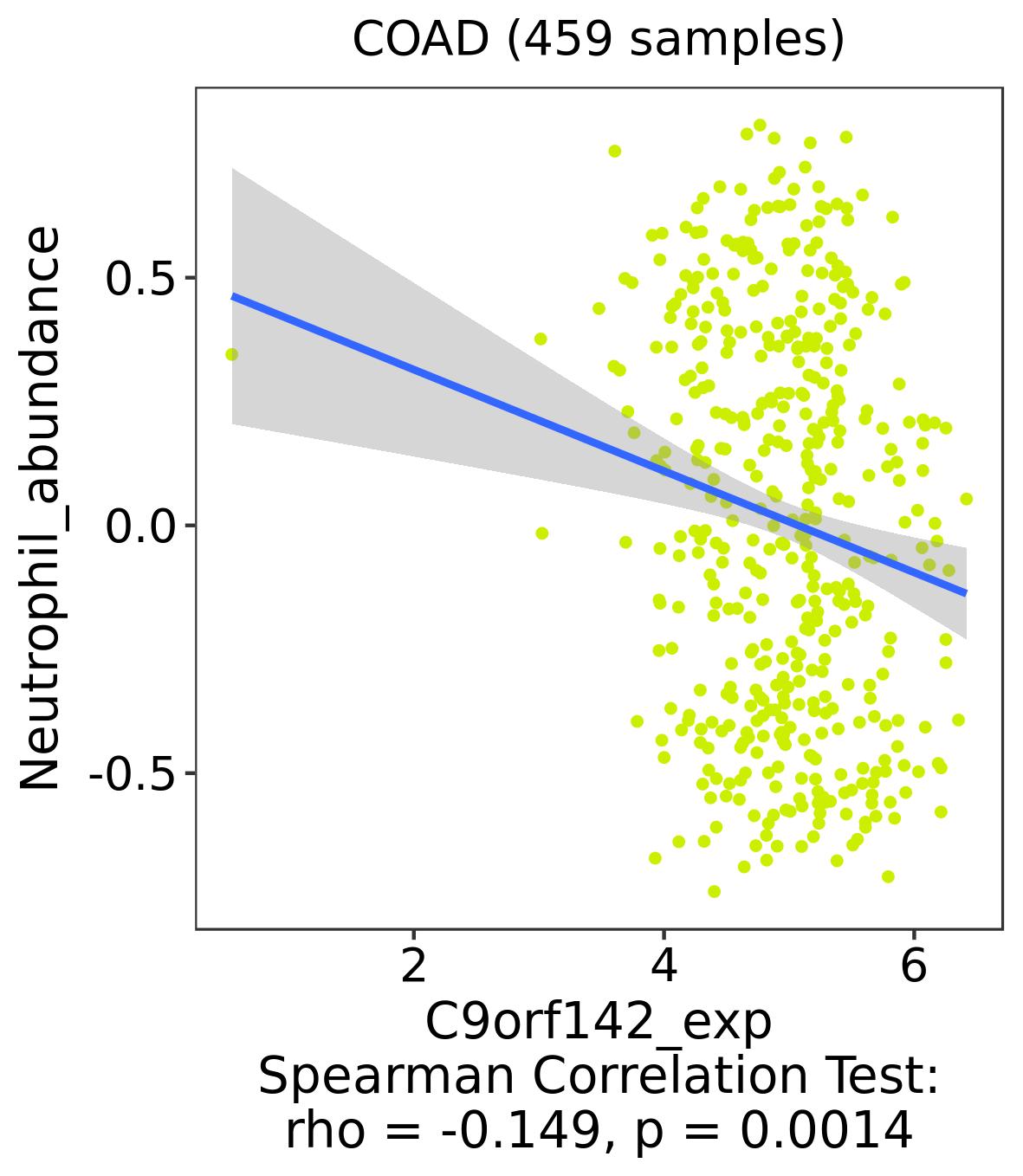

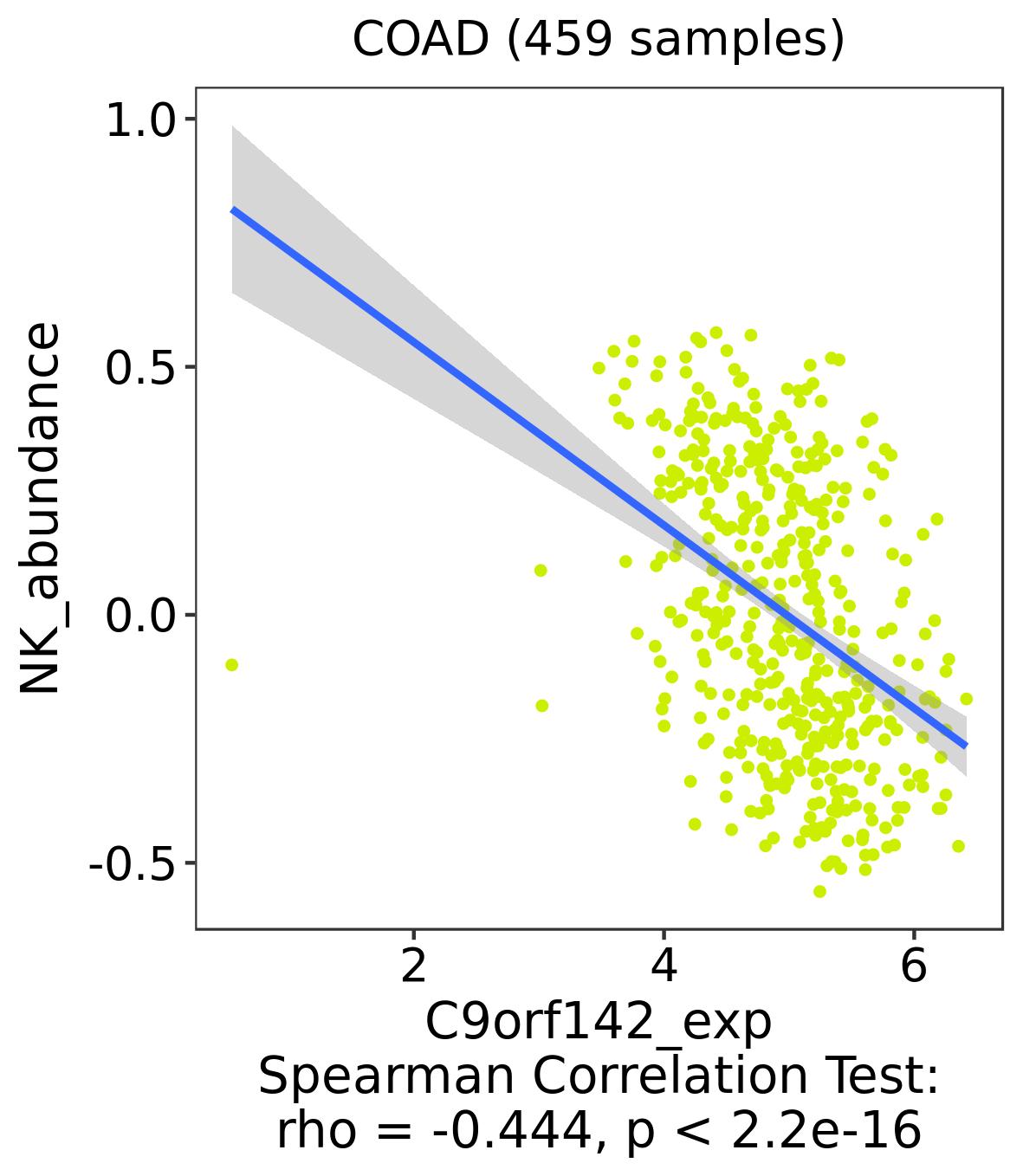

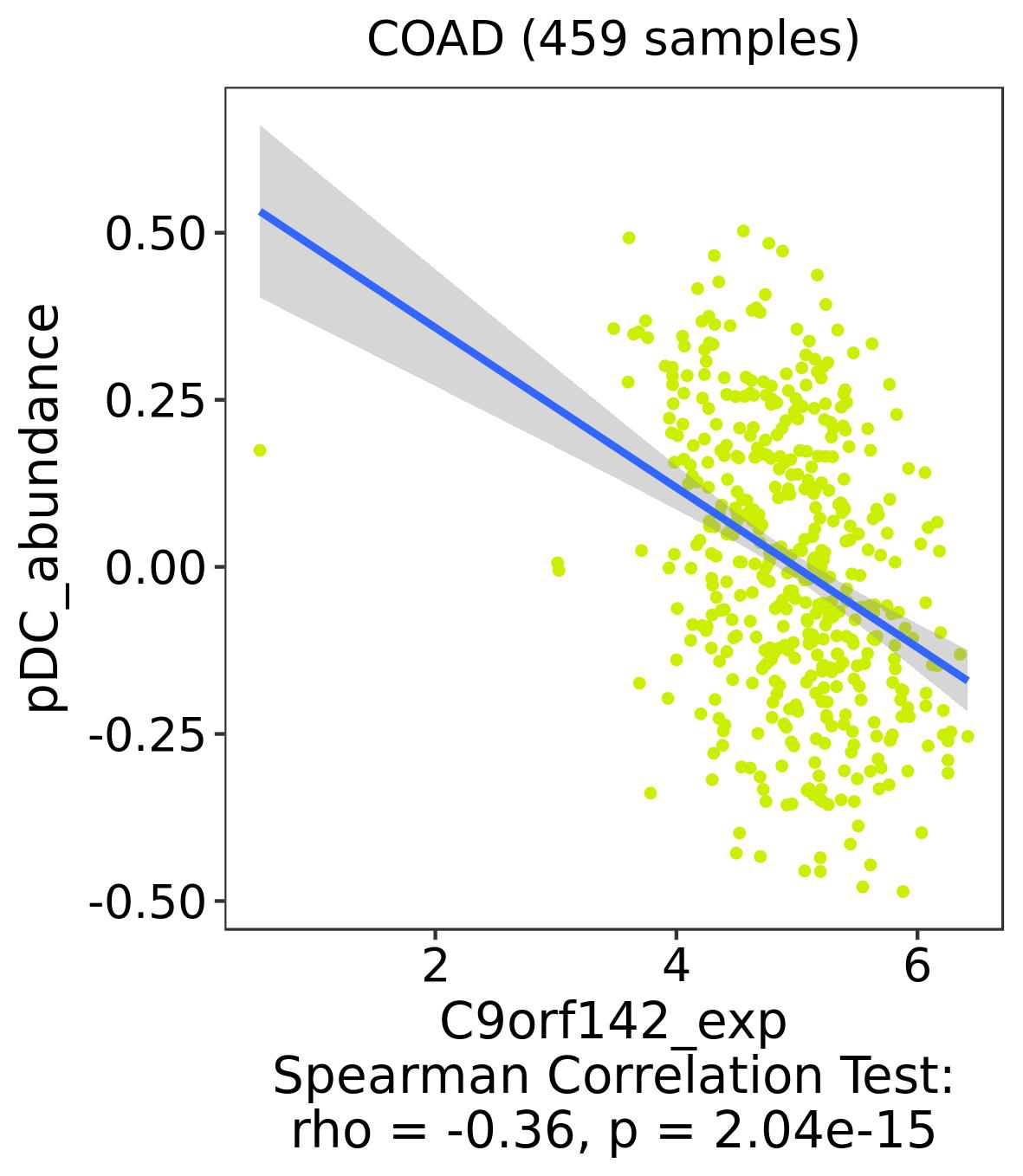

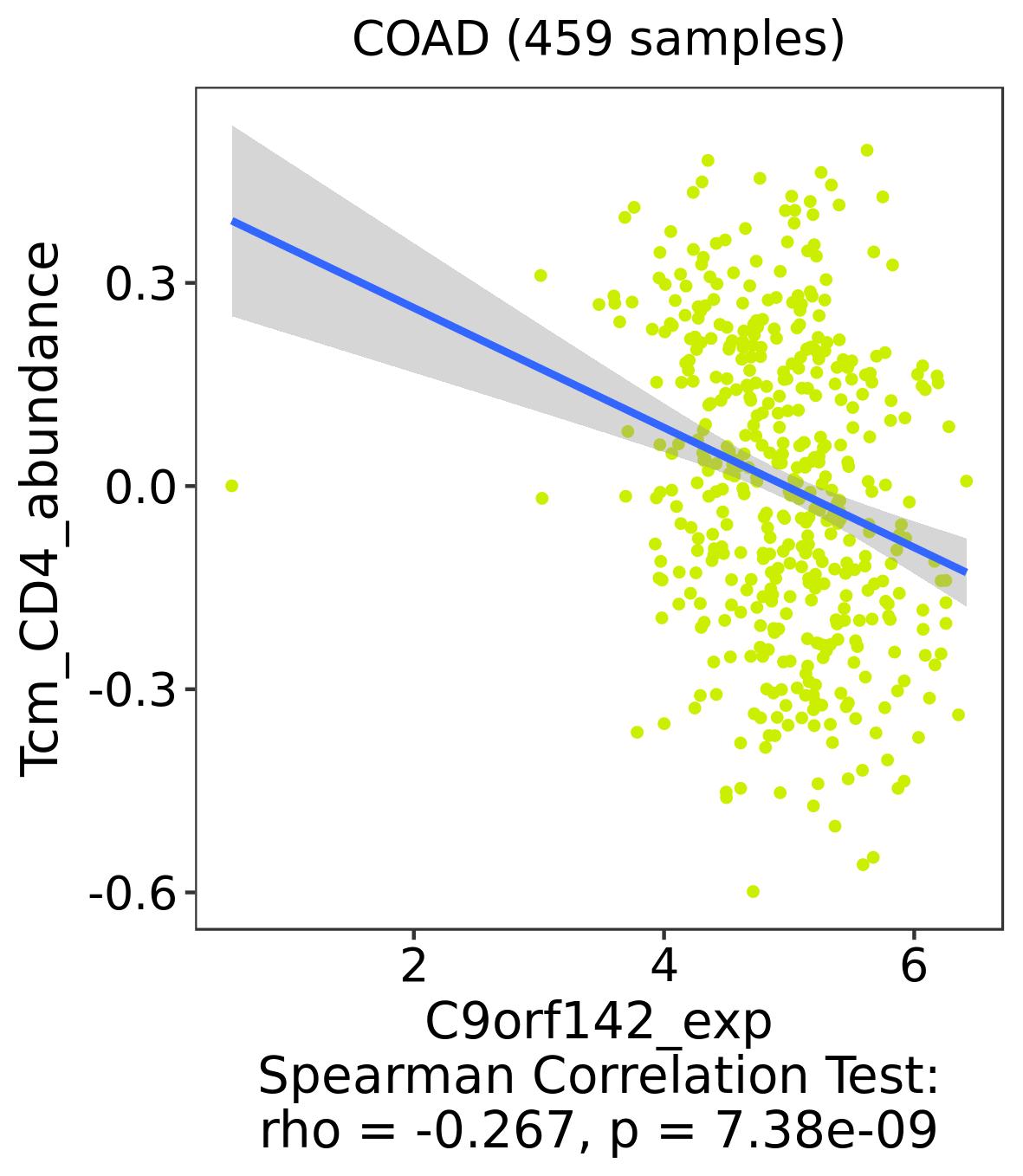

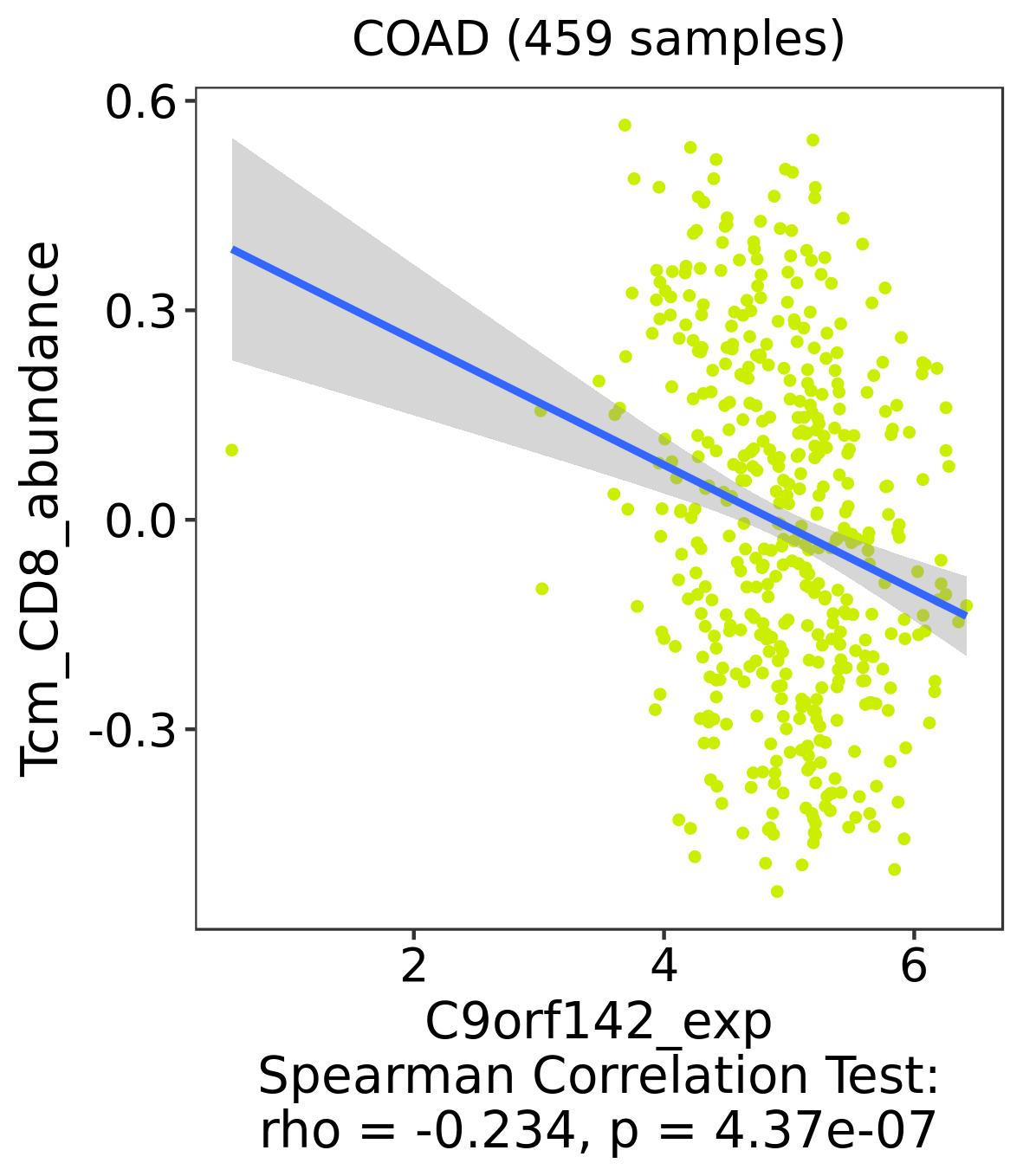

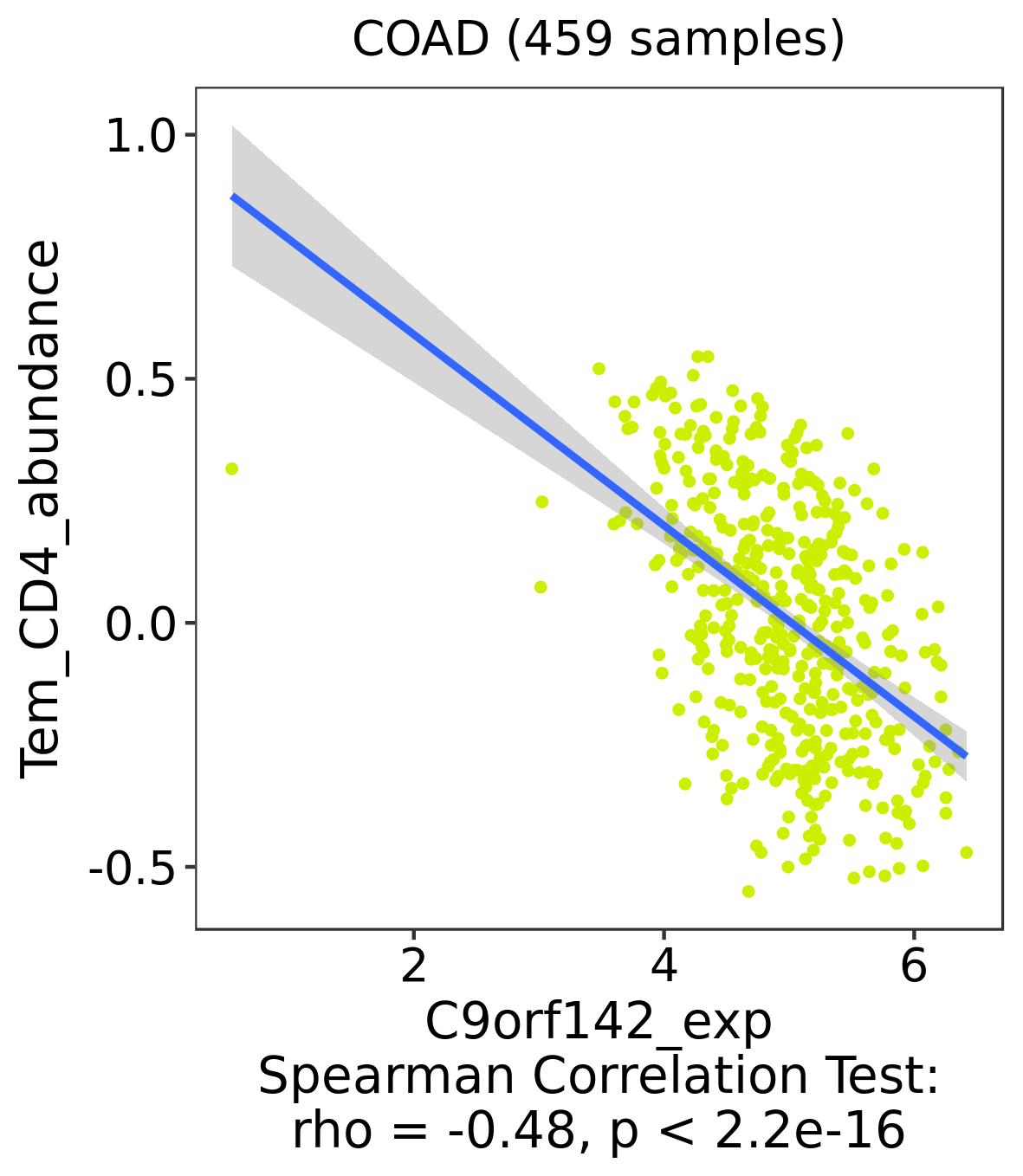

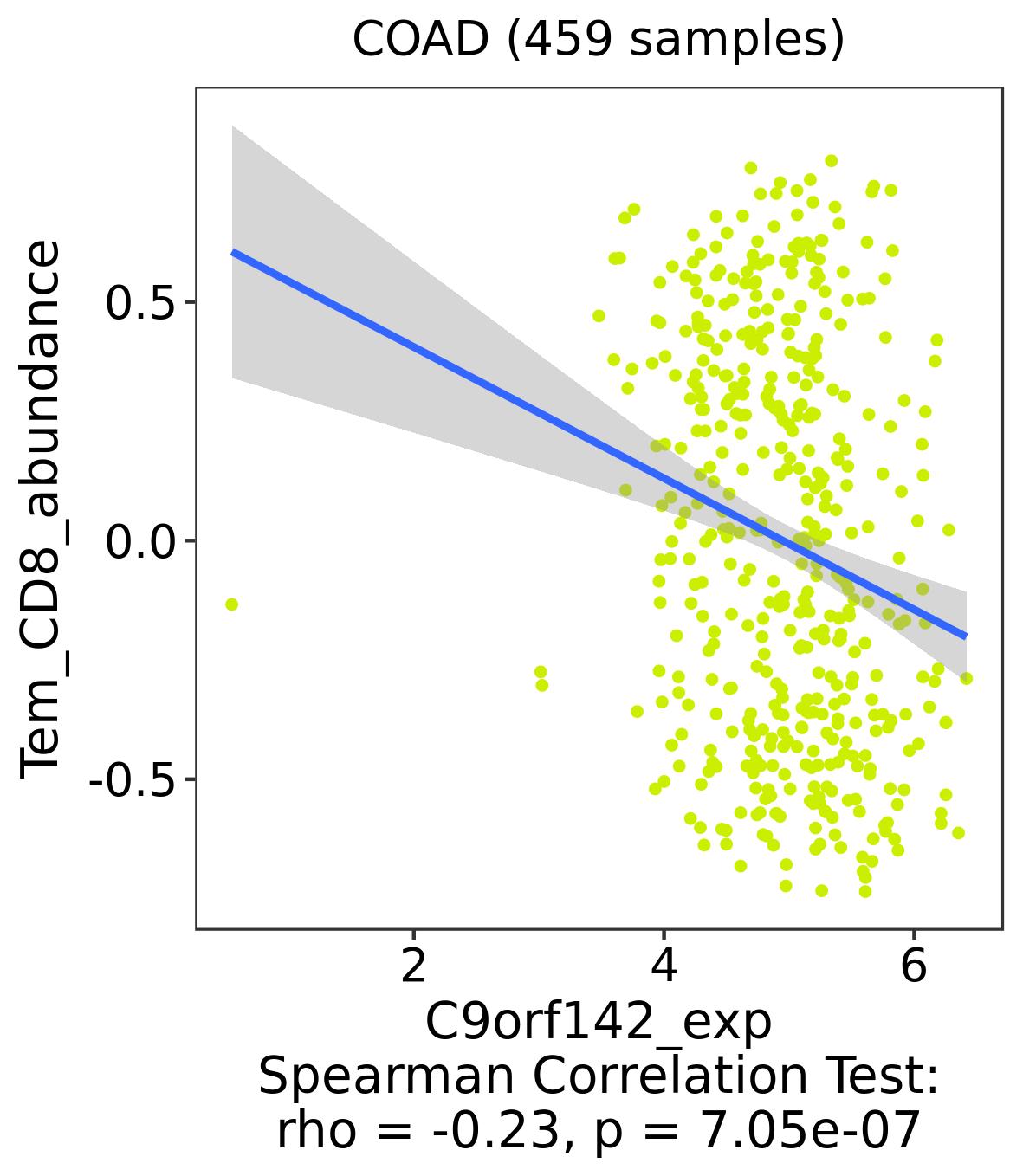


**Supplementary Figure S9:** Association of PAXX mRNA expression with 20 different immune cell types in TCGA-COAD dataset estimated from the TISIDB web server.
